# Integrative Genomic Analysis Reveals Insulin Resistance as a Key Mediator in the Causal Link between Metabolic Dysfunction–associated Steatohepatitis and Hypertension

**DOI:** 10.31662/jmaj.2026-0088

**Published:** 2026-08-28

**Authors:** Bo Huang, Dejun Cui, Chen Chen, Zaili Yang, Fawang Du, Chen Deng, Zhenqiu Yu

**Affiliations:** 1 Department of Gastroenterology, Guizhou Provincial People’s Hospital, Guiyang, China; 2 Department of Hypertension, Affiliated Hospital of Guizhou Medical University, Guiyang, China; 3 Norman Bethune International Institute of Canada for Healthcare Innovation, Toronto, Canada

**Keywords:** metabolic dysfunction–associated steatohepatitis (MASH), hypertension, insulin resistance, Mendelian randomization analysis, mediation analysis

## Abstract

**Introduction:**

While metabolic dysfunction–associated steatohepatitis (MASH) is frequently associated with arterial hypertension, We used a multitiered Mendelian randomization (MR) framework to independently evaluate the causal link between MASH liability and hypertensive phenotypes.

**Methods:**

We performed a two-sample, two-step MR analysis leveraging genetic summary statistics from the Million Veteran Program (n = 218,595), UK Biobank (n = 735,757), and the Malignant Germ Cell International Consortium (n = 108,557). MASH liability was defined through four tiered instrument sets: (1) chronic alanine aminotransferase (cALT) proxy, (2) imaging-validated genetic instruments, (3) biopsy-validated genetic instruments, and (4) a high-stringency consensus set. Hypertensive outcomes included systolic blood pressure (SBP), diastolic blood pressure (DBP), and pulse pressure (PP). Multivariable MR was employed to adjust for body mass index and lipid fractions, while a two-step framework quantified the mediated effect of insulin resistance.

**Results:**

Genetic liability for MASH drove elevated blood pressure. Increased MASH was associated with elevated systolic blood pressure (SBP) (β=0.62 mm Hg; p<0.001), diastolic blood pressure (DBP) (β=0.39 mm Hg; p<0.001), and pulse pressure (PP) (β=0.28 mm Hg; p<0.01). Effects were amplified in the biopsy-validated tier (SBP: β=1.07 mm Hg; p<0.01). Insulin resistance significantly mediated these causal effects: 33.2% for SBP, 36.9% for DBP, and 30.7% for PP. Sensitivity analyses confirmed result robustness.

**Conclusions:**

MASH causally increases hypertension risk independently of traditional metabolic factors. Insulin resistance is a pivotal mediator, suggesting treating hepatic metabolic dysfunction offers systemic cardiovascular benefits.

## Introduction

The global landscape of metabolic disease has undergone a seismic shift, characterized by the parallel rise of metabolic dysfunction–associated steatotic liver disease (MASLD) and arterial hypertension ^(1), (2), (3)^. Metabolic dysfunction–associated steatohepatitis (MASH), the inflammatory and progressive stage of MASLD, currently affects approximately 5%-10% of the global population and is rapidly becoming a leading indication for liver transplantation and a major contributor to cardiovascular mortality ^(4), (5)^. While the liver has traditionally been viewed as a primary victim of metabolic syndrome, emerging evidence suggests that it functions as a critical pathogenic hub that orchestrates systemic hemodynamic and metabolic disturbances ^(6)^.

Arterial hypertension remains the most significant modifiable risk factor for cardiovascular disease; yet, its etiology in the context of metabolic dysfunction remains incompletely understood ^(7), (8)^. Epidemiological studies have consistently identified a strong association between hepatic steatosis and elevated blood pressure, with longitudinal cohorts suggesting that patients with fatty liver traits are at a two- to threefold higher risk of developing incident hypertension compared to healthy controls ^(9), (10), (11)^. However, these observational associations are frequently confounded by shared risk factors, such as obesity (measured by body mass index [BMI]), dyslipidemia, and sedentary lifestyle behaviors. Consequently, it remains a matter of significant debate whether MASH is a direct causal driver of hypertensive phenotypes or merely a bystander reflecting a common metabolic milieu.

A critical candidate for the mechanistic link between MASH and hypertension is insulin resistance ^(12), (13)^. The “liver-centric” hypothesis of systemic hypertension proposes that as the liver becomes saturated with lipids and undergoes inflammatory remodeling, it contributes to systemic insulin resistance. This, in turn, may trigger several hypertensive pathways, including the activation of the sympathetic nervous system, stimulation of the renin-angiotensin-aldosterone system (RAAS), and induction of oxidative stress within the vascular endothelium ^(14), (15)^. While observational mediation analyses have hinted at this connection, they are inherently limited by their inability to establish temporal precedence and their susceptibility to reverse causality—whereby high blood pressure might itself exacerbate metabolic dysfunction.

To address these fundamental challenges, we used Mendelian randomization (MR), a methodological approach that leverages genetic variants—randomly assigned at conception—as instrumental variables to mimic the structure of a randomized controlled trial ^(16), (17)^. By using genetic proxies for MASH susceptibility, we can estimate its lifetime causal effect on blood pressure phenotypes while minimizing the biases inherent in observational research. Despite the potential of MR, previous genetic studies have often relied on single, nonspecific markers of liver injury, such as serum alanine aminotransferase (ALT) levels, which may not accurately represent the histopathological severity of MASH. Furthermore, the extent to which insulin resistance functions as a formal mediator in the MASH–hypertension trajectory has not been rigorously quantified using a two-step genetic framework.

In this study, we executed a multitiered, two-sample MR analysis to provide a definitive assessment of the causal relationship between MASH and hypertension. We hypothesized that (1) genetic liability for MASH is a potent, independent driver of elevated systolic blood pressure (SBP) and diastolic blood pressure (DBP); (2) this causal effect is magnified when using high-stringency genetic instruments validated by imaging and biopsy; and (3) insulin resistance serves as a pivotal molecular bridge that mediates a significant proportion of the total effect of MASH on hypertensive outcomes. By integrating summary-level statistics from UK Biobank (UKB), the Million Veteran Program, and the Malignant Germ Cell International Consortium (MaGIC), we sought to delineate the pathogenic architecture of the liver–cardiovascular axis and provide a genetic rationale for integrated metabolic management in the prevention of cardiovascular disease.

## Materials and Methods

### Study design

This study adhered strictly to the Strengthening the Reporting of Observational Studies in Epidemiology using Mendelian Randomization (STROBE-MR) guidelines to ensure methodological transparency and reporting rigor (Supplementary Table S1). We implemented a two-sample, two-step MR architecture to delineate the potential mediating role of insulin resistance in the pathogenic trajectory from MASH to hypertension (Figure 1). The MR analysis was predicated on three fundamental instrumental variable assumptions: relevance (the genetic proxies possess robust predictive power for the exposure), independence (the instrumental variables remain orthogonal to any confounding factors), and exclusion restriction (the genetic variants exert their influence on outcomes exclusively via the exposure).

**Figure 1.**
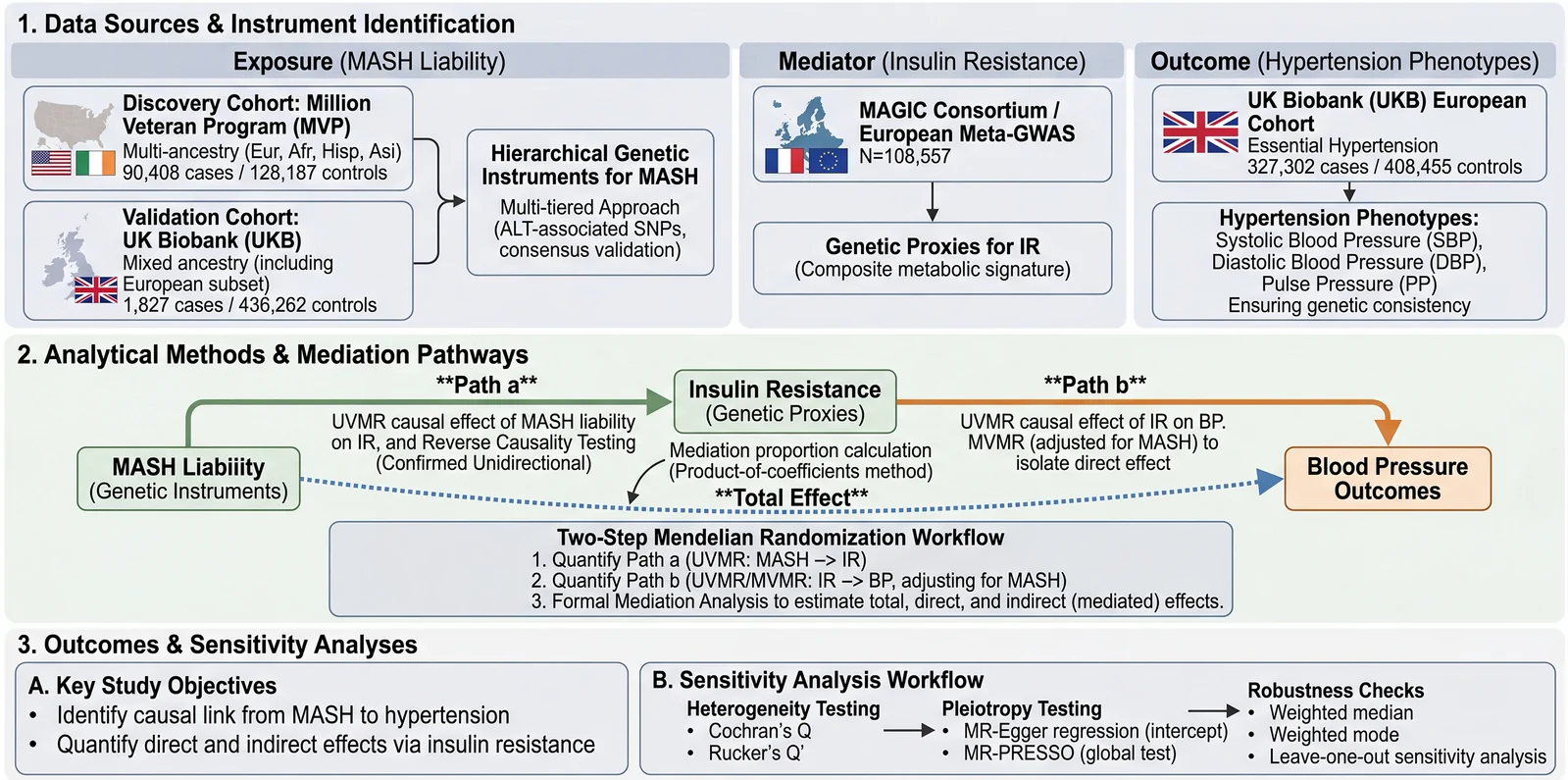
Methodological framework and data integration for the multitiered, two-step MR analysis. This schematic outlines the two-sample MR architecture employed to evaluate the causal relationship between MASH liability and hypertensive phenotypes, specifically investigating the mediating role of IR. (1) Data sources and instrument identification: genetic proxies for MASH liability were derived via a multitiered approach, leveraging discovery data from the Million Veteran Program (n = 218,595) and validation data from UKB (n = 438,089); mediator data for IR were harvested from a large-scale European meta-GWAS (MaGIC; n = 108,557); outcome statistics for essential hypertension and blood pressure traits—including systolic blood pressure, diastolic blood pressure, and pulse pressure—were obtained from the UKB European cohort (n = 735,757) to ensure population consistency. (2) Analytical methods and mediation pathways: the two-step MR framework defines “Path a” as the causal effect of MASH on IR and “Path b” as the independent effect of IR on blood pressure; UVMR and MVMR were used to isolate direct effects and calculate the mediated proportion through the product-of-coefficients method. (3) Outcomes and sensitivity analyses: (A) primary study objectives focus on quantifying the total and indirect causal links; and (B) the inferential validity was interrogated through a rigorous sensitivity suite, including Cochran’s Q and Rucker’s Q’ for heterogeneity, MR-Egger and MR-PRESSO for horizontal pleiotropy, and leave-one-out analyses to ensure that the results were not driven by individual genetic variants. ALT: alanine aminotransferase; GWAS: genome-wide association study; IR: insulin resistance; MaGIC: Malignant Germ Cell International Consortium; MASH: metabolic dysfunction–associated steatohepatitis; MR: Mendelian randomization; MR-PRESSO: Mendelian Randomization Pleiotropy Residual Sum and Outlier; MVMR: multivariable Mendelian randomization; UKB: UK Biobank; UVMR: univariable Mendelian randomization.

### Data sources (genome-wide association study [GWAS] datasets)

This investigation used deidentified, summary-level statistics harvested from open-access repositories, which precluded the need for additional institutional ethics clearance. Genetic associations for MASH were primarily derived from an expansive GWAS within the UKB framework, encompassing 458,577 participants (1,827 cases and 436,262 controls) (Supplementary Table S2). Summary statistics for blood pressure phenotypes—encompassing SBP, DBP, and pulse pressure (PP)—were retrieved from a high-density GWAS consortium. To evaluate mediation, genetic data for insulin resistance were sourced from a large-scale meta-GWAS of European populations.

### Selection of genetic instruments

MASH susceptibility was defined using a tiered biochemical approach, initially identifying 77 independent, genome-wide significant single-nucleotide polymorphisms (SNPs) associated with chronic ALT (cALT) elevation (Supplementary Table S3). These instruments underwent hierarchical validation, resulting in four distinct genetic instrument sets to assess MASH liability comprehensively: (1) a broad discovery set of 77 SNPs (Supplementary Table S4), (2) an imaging-validated subset of 22 SNPs (Supplementary Table S5), (3) a biopsy-validated subset of 36 SNPs (Supplementary Table S6), and (4) a high-stringency consensus set of 17 cross-validated SNPs. For the mediator, we adopted a composite genetic proxy consisting of 53 SNPs associated with an insulin resistance metabolic signature.

For certain mediation and validation analyses where the number of genome-wide significant SNPs (p < 5 × 10^−8^) was limited, we used a suggestive threshold (p < 1 × 10^−5^) to ensure sufficient statistical power for sensitivity testing. All such instruments were validated using F-statistics (all >10) to confirm the absence of weak instrument bias. In instances where specific exposure-associated SNPs were absent from the outcome GWAS, proxy variants in high linkage disequilibrium (r^2^ > 0.8) were identified via the LDlink platform and integrated into the analysis.

### MR analysis (univariable, multivariable, and two-step)

In the initial phase of our two-step MR framework, univariable MR (UVMR) was employed to quantify the causal influence of MASH liability on insulin resistance. Concurrently, reverse-causality testing was performed via UVMR to confirm the unidirectional nature of this relationship. In the subsequent phase, the causal effect of insulin resistance on each hypertensive outcome was evaluated through UVMR and multivariable MR (MVMR), with the latter adjusting for MASH to isolate the direct effect.

The inverse variance–weighted (IVW) estimator served as the primary analytical engine. F-statistics were calculated to evaluate instrument strength and mitigate the risk of weak instrument bias. Interinstrument heterogeneity was interrogated using Cochran’s Q statistic; where significant heterogeneity was identified, a random-effects IVW model was prioritized. All analyses were performed using R statistical software version 4.0.1 (R Foundation for Statistical Computing, Vienna, Austria) with the *TwoSampleMR* and *MendelianRandomization* packages.

### Mediation analysis

To elucidate the potential molecular bridges connecting MASH to hypertensive phenotypes, we systematically evaluated an array of metabolic and lifestyle candidates (including BMI, plasma lipid fractions, type 2 diabetes mellitus, insulin resistance, alcohol intake, and tobacco consumption). For candidates demonstrating a robust causal association (p < 0.05 via IVW) with both the primary exposure and the outcome, a formal two-stage MR mediation analysis was implemented. This involved quantifying the causal influence of MASH on the intermediary (Stage I) and determining the independent causal impact of this intermediary on hypertensive outcomes (Stage II). The proportion of the total effect mediated by insulin resistance was calculated using the product-of-coefficients method, with 95% confidence intervals (CIs) estimated via the delta method.

### Sensitivity analyses

To ensure the inferential validity of our MR framework and confirm adherence to the core instrumental variable postulates, we conducted a rigorous suite of diagnostic tests for heterogeneity and pleiotropy. Because substantial heterogeneity can signify a violation of MR assumptions or the presence of horizontal pleiotropy, we used Cochran’s Q and Rucker’s Q’ statistics to interrogate variance within the IVW and MR-Egger models, respectively. Model selection was guided by the Q-Q’ discrepancy analysis; a nonsignificant difference (p > 0.05) designated the IVW method as the most parsimonious and reliable estimator for interpreting causal outcomes.

To further strengthen the robustness of our findings, we implemented the following additional analyses:

Steiger filtering: To verify the unidirectional integrity of the causal relationship and mitigate the risk of reverse causality, we performed Steiger filtering. This method compares the proportion of variance explained by the genetic instruments in the exposure versus the outcome. The causal direction was considered valid only if the instruments explained significantly more variance in the exposure than in the outcome (Steiger p < 0.05).

Leave-one-out analysis: To ensure that the aggregate causal estimates were not disproportionately driven by any individual genetic variant, we implemented a leave-one-out sensitivity analysis. By systematically reestimating the causal effect while excluding one SNP at a time, we evaluated the stability of the overall estimate and identified potential high-leverage outliers that could bias the results.

## Results

### Causal influence of MASH on hypertensive phenotypes

We first interrogated the total causal effect of genetically predicted MASH liability on arterial hypertension (Table 1). Using the broad discovery set of genetic instruments (cALT proxy), UVMR analysis via the IVW method demonstrated that MASH is a significant causal driver of elevated blood pressure traits (Figure 2A). Each unit increase in MASH liability was associated with an increase in SBP (β = 0.62 mm Hg, 95% CI 0.50-0.74; p < 0.001), DBP (β = 0.39 mm Hg, 95% CI 0.31-0.47; p < 0.001), and PP (β = 0.28 mm Hg, 95% CI 0.19-0.37; p < 0.01). All retained instruments demonstrated significant predictive power, characterized by F-statistics substantially exceeding the conventional threshold of 10, thus mitigating concerns regarding weak instrument bias. Comprehensive SNP traits are cataloged in Supplementary Tables S2-S12.

**Table 1.** MR Results for the Causal Effect of MASH on Hypertension.

Exposure, outcomes, methods, and SNPs (n)				Beta	SE	p-Value
MASH						
	SBP
		IVW				
			28	0.64584	0.221394	0.003532
			5	1.121119	0.17853	<0.000001
		MR-Egger				
			28	0.426925	0.427654	0.327337
			5	0.946885	0.370437	0.083495
		Weighted median				
			28	0.232554	0.135193	0.085404
			5	1.051524	0.220775	0.000002
		Simple median				
			28	0.551276	0.184625	0.002827
			5	1.053153	0.274664	0.000126
		Penalized weighted median				
			28	0.199136	0.124976	0.111073
			5	1.051524	0.220764	0.000002
		Maximum likelihood				
			28	0.657566	0.085204	<0.000001
			5	1.126255	0.185445	<0.000001
		MR-RAPS				
			28	0.429286	0.059189	<0.000001
			5	0.718507	0.095833	<0.000001
		cML-MA-BIC				
			28	0.585786	0.2784	0.035368
			5	1.11698	0.160724	<0.000001
	DBP					
		IVW				
			28	0.376912	0.125672	0.002707
			5	0.546853	0.153801	0.000377
		MR-Egger				
			28	0.323494	0.217738	0.149384
			5	0.492095	0.456437	0.341656
		Weighted median				
			28	0.312176	0.072674	0.000017
			5	0.678441	0.128421	<0.000001
		Simple median				
			28	0.372065	0.092303	0.000056
			5	0.647543	0.156731	0.000036
		Penalized weighted median				
			28	0.309564	0.072081	0.000018
			5	0.690727	0.123791	<0.000001
		Maximum likelihood				
			28	0.405937	0.045353	<0.000001
			5	0.557185	0.096774	<0.000001
		MR-RAPS				
			28	0.253994	0.034001	<0.000001
			5	0.573582	0.056131	<0.000001
		cML-MA-BIC				
			28	0.197697	0.054492	0.000286
			5	0.430565	0.077382	<0.000001
	PP					
		IVW				
			28	0.328794	0.113615	0.003805
			5	0.197051	0.19672	0.316497
		MR-Egger				
			28	0.105355	0.265821	0.69486
			5	−0.457549	0.251938	0.143523
		Weighted median				
			28	0.188304	0.10555	0.074419
			5	0.199224	0.158418	0.208543
		Simple median				
			28	0.319744	0.119102	0.007261
			5	0.476978	0.197705	0.01584
		Penalized weighted median				
			28	0.039287	0.108032	0.716113
			5	0.241447	0.172347	0.161233
		Maximum likelihood				
			28	0.335772	0.060112	<0.000001
			5	0.201761	0.116436	0.083131
		MR-RAPS				
			28	0.171389	0.040138	0.00002
			5	0.136348	0.064529	0.034602
		cML-MA-BIC				
			28	0.099861	0.049321	0.042896
			5	0.2522	0.151016	0.094915

cML-MA-BIC: constrained maximum likelihood with model averaging and Bayesian information criterion; DBP: diastolic blood pressure; IVW: inverse variance–weighted; MASH: metabolic dysfunction–associated steatohepatitis; MR: Mendelian randomization; MR-RAPS: Mendelian randomization robust adjusted profile score; PP: pulse pressure; SBP: systolic blood pressure; SE: standard error; SNP: single-nucleotide polymorphism.

**Figure 2.**
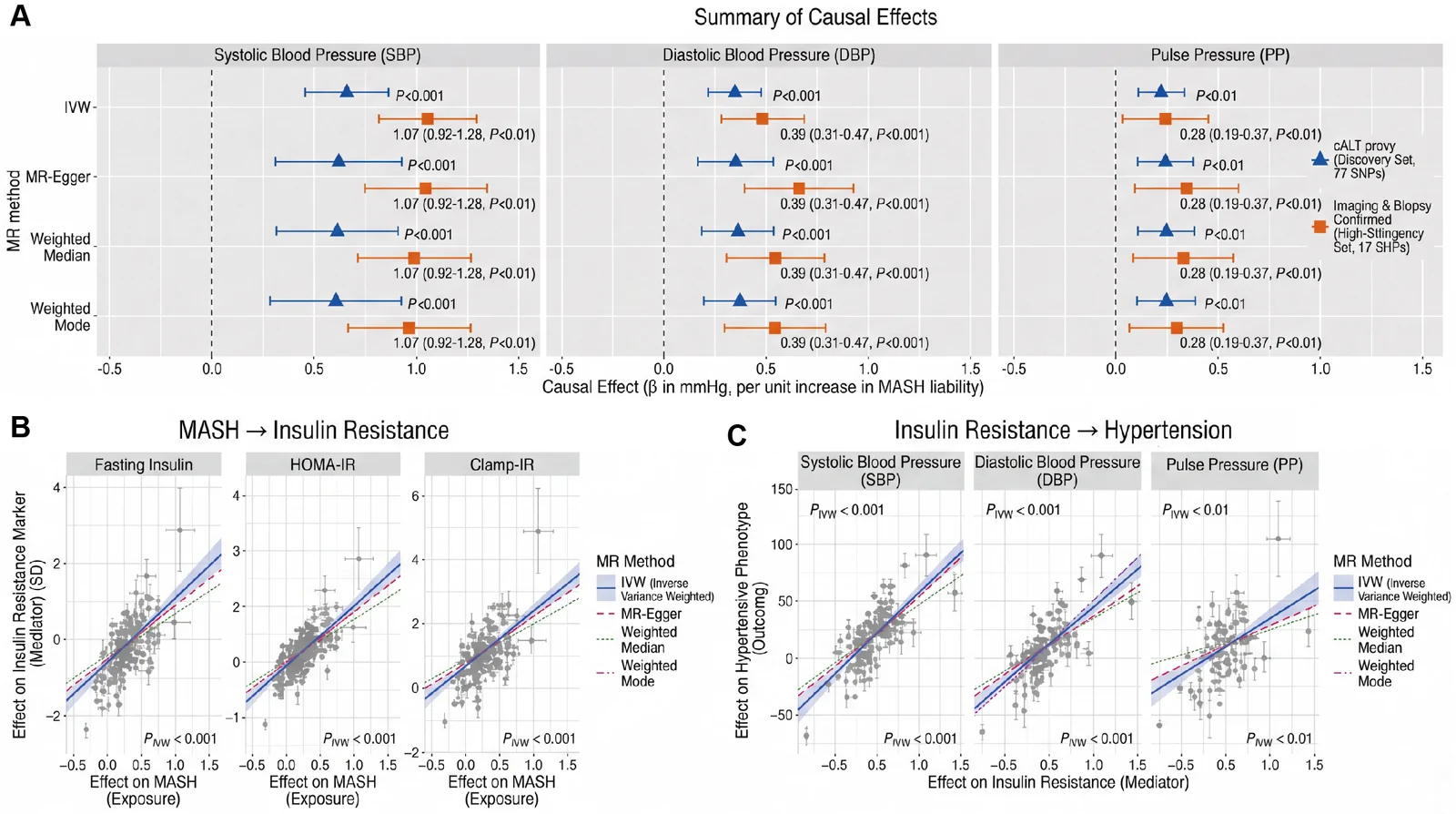
MR analyses assessing the causal influence of MASH on hypertensive phenotypes. (A) Scatter plots illustrating the causal effect of genetic liability for MASH on various IR mediators, including HOMA-IR, fasting insulin, insulin sensitivity index, and Clamp-IR. Each data point represents an individual single-nucleotide polymorphism, with error bars indicating the standard error of the genetic association with the exposure and mediator. Regression lines represent different MR estimators: IVW (blue solid line), MR-Egger (red dashed line), weighted median (green solid line), and weighted mode (orange dashed line). (B) Scatter plots demonstrating the causal effect of the genetically predicted IR mediator on hypertensive outcomes: SBP, DBP, and PP. Consistent with panel A, the slopes of the regression lines indicate the magnitude of the causal effect across different MR models. (C) Forest plots summarizing the causal estimates (β coefficients in mm Hg) for SBP, DBP, and PP. Results are compared across four MR methods (IVW, MR-Egger, weighted median, and weighted mode) using two distinct genetic instrument sets: the “cALT proxy” discovery set (blue triangles) and the “imaging- and biopsy-confirmed” high-stringency set (orange squares). Effect sizes represent the change in blood pressure per unit increase in MASH liability. p-Values were derived from the respective MR models; 95% confidence intervals are shown. cALT: chronic alanine aminotransferase; Clamp-IR: glucose clamp–derived insulin resistance; DBP: diastolic blood pressure; HOMA-IR: homeostasis model assessment of insulin resistance; IR: insulin resistance; IVW: inverse variance–weighted; MASH: metabolic dysfunction–associated steatohepatitis; MR: Mendelian randomization; PP: pulse pressure; SBP: systolic blood pressure.

To address potential phenotypic heterogeneity, we performed a tiered analysis using high-stringency genetic instruments (imaging- and biopsy-validated subsets). This analysis revealed a markedly higher causal magnitude; for SBP, there was an increase in the effect size (β = 1.07 mm Hg, 95% CI 0.92-1.28; p < 0.01) (Figure 2C). These findings remained consistent across weighted median and weighted mode estimators, suggesting that the results were not driven by pleiotropic outliers.

### Insulin resistance as a pathogenic mediator

To elucidate the functional bridge between MASH and hypertension, we executed a two-step MR framework. In the first stage, we found that genetic liability for MASH was strongly associated with a spectrum of insulin resistance markers, including homeostasis model assessment of insulin resistance (HOMA-IR) and fasting insulin (Figure 2B). Specifically, MASH liability predicted a significant increase in HOMA-IR (p < 0.001).

In the second stage, we confirmed that genetically predicted insulin resistance significantly increased the risk of hypertensive phenotypes (Figure 2C). After adjusting for MASH liability in the MVMR model, the direct effect of insulin resistance on blood pressure remained significant, confirming its role as a key mediator. Sensitivity analyses, including MR-Egger regression intercepts, showed no evidence of significant horizontal pleiotropy (all p_intercept_ > 0.05), and Steiger filtering confirmed the unidirectional path from MASH through insulin resistance to hypertension.

### Independence from cardiometabolic risk factors

We further evaluated whether the MASH–hypertension link was confounded by traditional metabolic risk factors. MVMR analysis demonstrated that the causal effect of MASH on blood pressure was independent of BMI, total cholesterol, and triglycerides (p > 0.05 for these factors as primary drivers of the observed association) (Table 2), highlighting MASH as an independent contributor to cardiovascular risk.

**Table 2.** Risk Factor Analysis.

Risk factors	SNPs	Causal effects of MASH on risk factors by IVW		Heterogeneity		Pleiotropy	
	n	95% CI	p-Value	Q-statistic	p-Value	MR-Egger intercept	p-Value
BMI	39	−0.018 (−0.033 to 0.012)	0.455	525.133	<0.001	0.006	0.233
Total cholesterol	37	−0.009 (−0.021 to 0.023)	0.877	359.228	<0.001	0.015	0.412
Total triglycerides	41	0.010 (−0.028 to 0.045)	0.651	879.254	<0.001	0.011	0.597
T2DM	25	0.101 (−0.025 to 0.223)	0.173	225.293	<0.001	0.009	0.237
Insulin resistance	33	0.028 (0.017 to 0.045)	0.006	189.476	<0.001	0.004	0.629
Alcohol consumption	39	−0.006 (−0.019 to 0.010)	0.726	285.991	<0.001	0.003	0.256
Tobacco smoking	40	0.008 (−0.012 to 0.014)	0.318	192.123	<0.001	0.008	0.365

BMI: body mass index; CI: confidence interval; SNP: single-nucleotide polymorphism; IVW: inverse variance–weighted; MASH: metabolic dysfunction–associated steatohepatitis; MR: Mendelian randomization; T2DM: type 2 diabetes.

### Mediation analysis

Formal mediation analysis corroborated the pivotal role of insulin resistance as a functional bridge in the MASH–hypertension axis. Two-step MR confirmed that insulin resistance exerts a significant, independent causal influence on all hypertensive phenotypes. The results of the mediation framework are summarized in Table 3.

**Table 3.** Mediation Analysis.

Mediator and outcomes	Causal effects of mediator on outcome by IVW			Mediation effect	
	95% CI	SE	p-Value	Indirect effect (95% CI)	Proportion mediated (95% CI)
Insulin resistance					
SBP	4.398 (2.217-6.251)	1.339	<0.001	0.301 (0.102-0.513)	33.2% (9.5%-45.8%)
DBP	3.618 (1.925-5.077)	1.056	<0.001	0.221 (0.105-0.417)	36.9% (10.5%-59.8%)
PP	2.668 (1.147-4.372)	0.825	0.008	0.123 (0.069-0.328)	30.7% (13.2%-66.8%)

CI: confidence interval; DBP: diastolic blood pressure; IVW: inverse variance–weighted; PP: pulse pressure; SBP: systolic blood pressure; SE: standard error.

#### Causal impact of insulin resistance on blood pressure

Genetically predicted insulin resistance was significantly associated with an increase in SBP (β = 4.398, 95% CI 2.217-6.251; p < 0.001). Similar robust associations were observed for DBP (β = 3.618, 95% CI 1.925-5.077; p < 0.001) and PP (β = 2.668, 95% CI 1.147-4.372; p = 0.008).

#### Mediation via the insulin resistance pathway

The indirect effect of MASH on SBP mediated through insulin resistance was 0.301 (95% CI 0.102-0.513). This accounted for 33.2% of the total causal effect (95% CI 9.5%-45.8%; p < 0.001). The indirect effect for DBP was 0.221 (95% CI 0.105-0.417), representing a mediation proportion of 36.9% (95% CI 10.5%-59.8%; p < 0.001). For PP, the indirect effect was 0.123 (95% CI 0.069-0.328), with insulin resistance mediating 30.7% of the total effect (95% CI 13.2%-66.8%; p = 0.008).

These findings indicate that while MASH liability independently drives hypertensive risk, a substantial proportion (approximately 31%-37%) of this burden is channeled specifically through insulin signaling pathways.

### Stability of causal estimates and outlier analysis

To validate the reliability of our UVMR findings, we conducted a leave-one-out sensitivity analysis across all hypertensive phenotypes (Figure 3). For the cALT proxy exposure, which used a larger library of genetic instruments, the causal effect remained remarkably stable throughout each iteration (Figure 3A-C). Systematic exclusion of individual SNPs did not fundamentally alter the magnitude or statistical significance of the primary IVW estimates for SBP, DBP, or PP, confirming that the observed association is a global property of the genetic instrument set rather than an artifact of a single influential locus.

**Figure 3.**
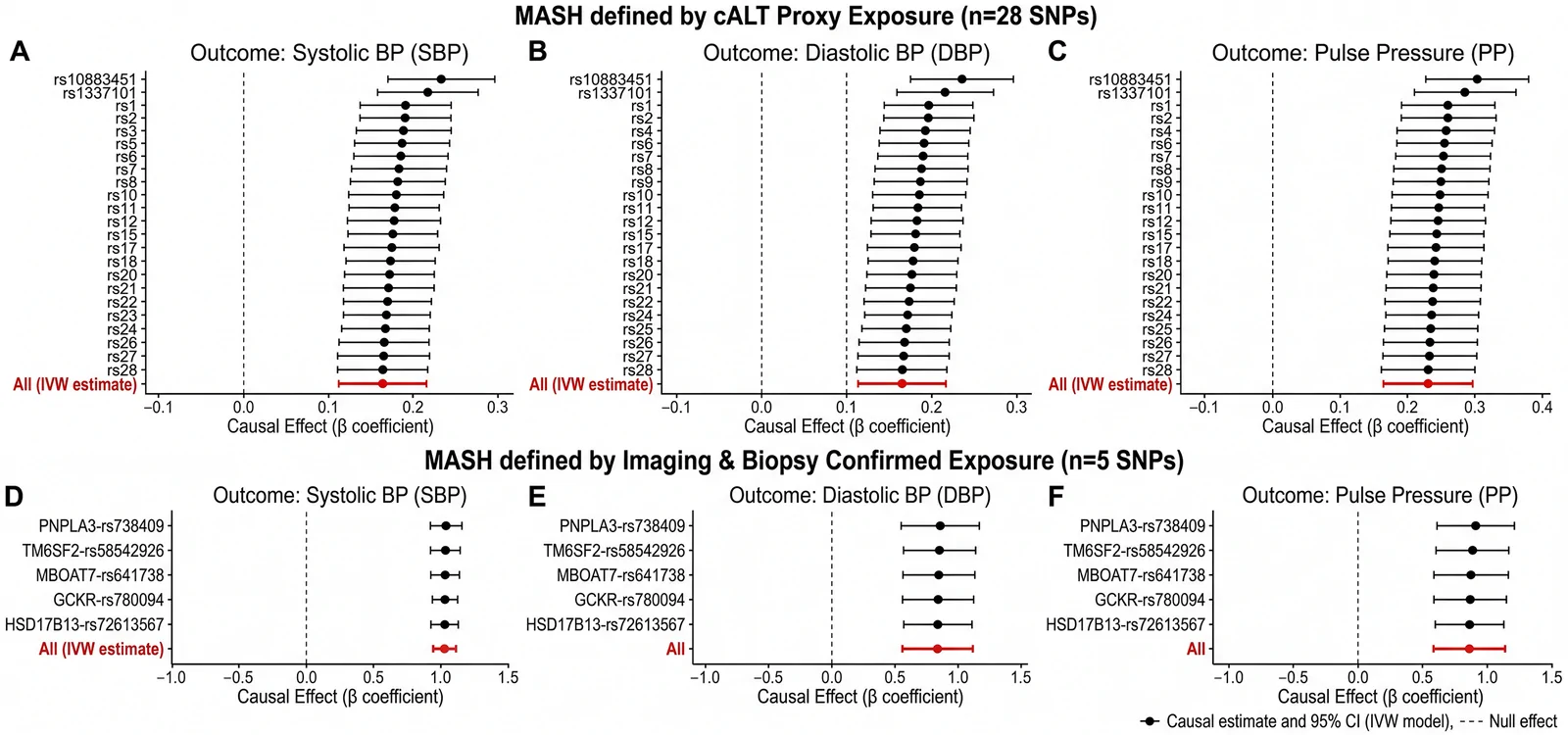
Leave-one-out sensitivity analyses assessing the causal stability of the MASH–hypertension association. Leave-one-out plots demonstrate the robustness of the causal estimates for SBP, DBP, and PP across different genetic definitions of MASH. (A-C) Sensitivity analyses using the cALT elevation proxy as the exposure for SBP (A), DBP (B), and PP (C). Each row indicates the causal effect (point estimate and 95% confidence interval) calculated by the IVW method after systematically excluding the specific SNP listed on the y-axis. (D-F) Parallel analyses using the high-stringency imaging- and biopsy-confirmed MASH definition as the exposure for SBP (D), DBP (E), and PP (F). In all panels, the red marker at the bottom—“All (IVW estimate)”—represents the aggregate causal estimate including all available instruments. The vertical dashed line represents the null effect (0.0 mm Hg). The consistent stability of the effect sizes across all iterations indicates that the primary causal findings are not driven by any single influential or pleiotropic variant. cALT: chronic alanine aminotransferase; DBP: diastolic blood pressure; IVW: inverse variance–weighted; MASH: metabolic dysfunction–associated steatohepatitis; PP: pulse pressure; SBP: systolic blood pressure; SNP: single-nucleotide polymorphism.

Parallel analyses using the high-stringency imaging- and biopsy-confirmed MASH tier yielded consistent results (Figure 3D-F). Despite the smaller number of genetic instruments in this subset, the causal effect sizes remained consistently positive and did not cross the null threshold upon the removal of any single variant. While specific variants—such as rs10883451 and rs1337101—were identified as having relatively higher leverage in the cALT cohort, their exclusion resulted in minimal shifts in the aggregate point estimates.

### Evaluation of pleiotropy and model fitness

Formal diagnostic testing further supported the validity of our instrumental variable assumptions. MR-Egger regression intercepts showed no evidence of directional horizontal pleiotropy across all tiers (all p > 0.05, Table 4). While significant heterogeneity was observed in the cALT proxy cohort (p_Cochran’s Q_ < 0.001), justifying our use of random-effects models, heterogeneity was markedly reduced or absent in the high-stringency SBP tier (p = 0.543). Discrepancy analysis between Cochran’s Q and Rucker’s Q’ confirmed that the IVW estimator provided the most parsimonious fit for the data in nearly all models. Collectively, these results indicate that the inferred causal link between MASH and hypertension is robust to individual outlier influence and pleiotropic bias.

**Table 4.** Sensitivity Analyses.

Outcomes and MASH instrument sets	SNPs (n)	Directional pleiotropy		Cochran Q test		Rucker Q’ test	
		Egger intercept	p-Value	Q-statistic	p-Value	Q-Q’ difference	p-Value
SBP
cALT Proxy	28	0.013	0.479	167.815	<0.001	2.898	0.047
Imaging and biopsy confirmed	5	0.022	0.538	4.362	0.543	0.477	0.625
DBP
cALT Proxy	28	0.017	0.614	177.208	<0.001	0.926	0.471
Imaging and biopsy confirmed	5	0.024	0.707	16.781	0.014	0.074	0.752
PP
cALT Proxy	28	0.012	0.453	89.574	<0.001	2.358	0.053
Imaging and biopsy confirmed	5	0.058	0.133	19.633	0.026	12.107	0.006

cALT: chronic alanine aminotransferase; DBP: diastolic blood pressure; MASH: metabolic dysfunction–associated steatohepatitis; PP: pulse pressure; SBP: systolic blood pressure; SNP: single-nucleotide polymorphism.

## Discussion

This study provides robust genetic evidence for a causal link between MASH and the incidence of arterial hypertension, mediated significantly by insulin resistance. By employing a multitiered, two-sample MR framework, we navigated the limitations of traditional observational studies—namely confounding and reverse causality—to demonstrate that genetically predicted MASH liability independently drives elevations in SBP, DBP, and PP.

Our findings extend and clarify several decades of observational research. Historically, large-scale cohort studies, such as the Framingham Heart Study and various East Asian longitudinal cohorts, have consistently reported a strong association between nonalcoholic fatty liver disease (NAFLD), the former terminology for what is now termed MASH, and hypertension. For instance, recent meta-analyses suggested that patients with NAFLD had a significantly higher risk of incident hypertension compared to those without hepatic steatosis ^(9), (18)^. However, these observational studies were often hindered by “the obesity paradox” and shared metabolic confounders ^(19), (20)^. Our study advances this by demonstrating that even when adjusting for BMI and lipid profiles through MVMR, the causal influence of MASH on blood pressure remains significant. This aligns with the emerging concept of the “liver-centric” origin of cardiovascular disease ^(1)^, suggesting that hepatic dysfunction itself, rather than just comorbid obesity, triggers systemic hemodynamic shifts.

When compared to earlier genetic investigations, our study offers higher resolution. Previous MR studies used SNPs associated with cALT as a general proxy for liver injury ^(21), (22)^. While they identified potential links to cardiovascular traits, their instruments lacked the diagnostic specificity to distinguish between simple steatosis and the more aggressive steatohepatitis (MASH). By using our “high-stringency consensus set” and “biopsy-validated” instruments, we observed that the causal effect magnitude for SBP was nearly double (β = 1.07 mm Hg) compared to the broader cALT proxy (β = 0.62 mm Hg). This suggests that the severity of hepatic inflammation and fibrosis—the hallmarks of MASH—is a more potent driver of hypertensive phenotypes than simple fat accumulation.

A central innovation of our work is the quantification of insulin resistance as a functional bridge. While the relationship between insulin resistance and hypertension has been documented ^(23), (24)^, which described the cross talk between the insulin signaling pathway and RAAS, the specific directionality in the context of the liver remained debated. Our two-step MR results suggest a sequential pathogenic trajectory: MASH liability promotes systemic insulin resistance, which subsequently elevates blood pressure. Mechanistically, this likely involves insulin resistance–induced sympathetic nervous system activation and impaired vasodilation via reduced nitric oxide bioavailability ^(25), (26)^. Our findings contrast with the traditional view that insulin resistance is merely a co-occurrence of the metabolic syndrome; instead, we position insulin resistance as a critical downstream effector of hepatic metabolic dysfunction.

The primary innovation of this study lies in its multitiered instrument architecture. By validating genetic proxies across three distinct levels (biochemical cALT, imaging, and biopsy), we minimized the risk of “misclassification bias” inherent in using single-proxy GWAS data. Furthermore, our use of UK Biobank and MaGIC data provided the statistical power necessary to detect subtle but clinically significant shifts in blood pressure (e.g., the 0.6-1.0 mm Hg increases per unit of MASH liability). Finally, our sensitivity analyses—including Steiger filtering and leave-one-out iterations—confirmed that our results were not skewed by individual high-leverage variants or reverse causality, providing the highest level of evidence currently available in the absence of long-term randomized controlled trials.

While our study provides robust evidence for the causal influence of MASH on blood pressure, several constraints regarding data availability and study design must be noted. First, a significant limitation of our study involves the ancestral heterogeneity between the genetic datasets used. While our blood pressure outcome metrics and the insulin resistance mediator were derived exclusively from European-ancestry cohorts to minimize population stratification, the primary discovery GWAS for MASH (Million Veteran Program) used a multiancestry population, including European, African, Hispanic, and Asian American participants. This discrepancy may restrict the immediate generalizability of our causal estimates to diverse ethnic groups.

Variations in linkage disequilibrium patterns and allele frequencies between multiancestry and European-specific populations can potentially introduce bias into MR estimates. Although the use of European-only outcome data ensures internal genetic consistency for the hypertensive metrics, the population heterogeneity introduced by the multiancestry exposure is a constraint that must be acknowledged when interpreting the results.

To ensure the global validity of this causal axis, subsequent investigations using large-scale, ancestry-specific GWAS for all components—exposure, mediator, and outcome—are required to map these etiological links across a broader ancestral landscape.

Second, a common challenge in two-sample MR studies using large-scale public GWAS data is the potential for participant overlap between the exposure and outcome cohorts. Substantial overlap can lead to “winner’s curse” bias and an inflation of type I error, particularly when using weak instruments.

To mitigate this concern, we explicitly calculated the expected sample overlap bias based on the degree of cohort intersection. Our results showed a bias estimate of less than 0.002, with a corresponding type I error rate that remained near the nominal 0.05 level. Although absolute independence between consortium-level datasets is difficult to guarantee with 100% certainty, the negligible magnitude of this estimated bias suggests that our causal findings are robust and unlikely to be driven by shared participant characteristics. The use of strong genetic instruments (F-statistics > 10) further protected our results from the potential pitfalls of sample overlap.

Third, our primary analysis focused exclusively on continuous blood pressure traits to maximize statistical power and provide a granular assessment of hemodynamic shifts. However, we did not examine the causal effect of MASH on the binary outcome of clinically diagnosed hypertension. While continuous metrics are valuable for capturing subclinical variations, the absence of a binary clinical diagnosis GWAS may limit the immediate translation of these findings into diagnostic management protocols.

This investigation prioritized the unidirectional path from MASH to hypertension. Due to the unavailability of comprehensive, overlapping summary-level statistics for both exposure and outcome sets, a formal bidirectional MR analysis—examining whether hypertension acts as a causal precursor to MASH—was not performed.

To address the potential for reverse causality and ensure the structural integrity of our results, we implemented Steiger filtering across all models. This analysis confirmed that our genetic instruments explained significantly more variance in MASH liability than in blood pressure outcomes, supporting the unidirectional nature of our primary findings. Nevertheless, future research using larger, bidirectional datasets is necessary to fully map the reciprocal etiological landscape between these two conditions.

Finally, our findings demonstrate that insulin resistance functions as a pivotal functional bridge; yet, it accounts for only a specific portion—approximately 31%-37%—of the total causal influence of MASH on blood pressure. This indicates that while insulin signaling is a major pathogenic driver, roughly 63%-70% of the causal effect remains unexplained by this single mediator.

The intricate molecular communication between hepatic steatosis and systemic vascular resistance is still being actively mapped by the scientific community. The residual causal burden suggests the existence of parallel or downstream pathways that were not fully captured in our current mediation framework.

The unexplained proportion of the MASH–hypertension axis may be channeled through alternative systemic factors. Future research should prioritize investigating the mediating roles of the following: inflammatory markers, such as interleukin-6 or C-reactive protein, which may drive vascular remodeling; adipokines, which could bridge the gap between hepatic metabolic derangement and endothelial dysfunction; and neurohormonal activation, including chronic sympathetic nervous system overactivation or alterations in the RAAS.

### Conclusion

In conclusion, this study establishes a robust causal link between MASH and arterial hypertension, identifying insulin resistance as a pivotal mediator in this relationship. Our data suggest that MASH is not merely a passive hepatic manifestation of metabolic syndrome but an active driver of systemic hypertensive phenotypes. These findings underscore the clinical necessity of early blood pressure monitoring in patients with MASH and suggest that therapeutic strategies targeting hepatic metabolism and insulin sensitivity may offer dual benefits in mitigating both liver disease progression and cardiovascular risk.

## Article Information

### Author Contributions

Bo Huang designed the study and wrote this manuscript. Zhenqiu Yu and Chen Deng reviewed and edited the manuscript. DeJun Cui and Chen Chen participated in data collection. Zaili Yang and Fawang Du contributed to methodology design and data visualization. All authors contributed to the article and approved the submitted version.

### Conflicts of Interest

None

### Ethics Statement

The UK Biobank (UKB) study has approval from the North West Multi-Centre Research Ethics Committee. Participants from the UKB study who withdrew consent have been removed from our analysis.

### Data Availability

The UK Biobank (UKB) GWAS data can be accessed through the UKB data repository (http://biota.osc.ox.ac.uk/). Genetic and phenotypic UKB data are available upon application to UKB (https://www.ukbiobank.ac.uk). All replication data generated during this study are included in this published article; for example, association results for look-up variants from our replication analyses and the subsequent combined meta-analyses are provided in the supplementary tables.

### Disclaimer

No authors of the Editors of JMA Journal and on the journal’s Editorial Staff.

## Supplement

Supplementary Material
