# Supplementary material for: Integrative Genomic Analysis Reveals Insulin Resistance as a Key Mediator in the Causal Link between Metabolic Dysfunction–associated Steatohepatitis and Hypertension
Source: JMA J. 2026 Aug 28;9(5):1164–75. doi: 10.31662/jmaj.2026-0088 (PMC13639412; doi:10.31662/jmaj.2026-0088)
Supplement: Supplementary file 1 [file 2433-3298-9-5-1164-s001.pdf]

SUPPLEMENTARY MATERIALS

Table S1. STROBE-MR Checklist of Recommended Items to Address in Reports of Mendelian Randomization Studies*

Item No.	Section	Checist item	Page
Title and Abstract			
1	Title and abstract	Indicate mendelian randomization (MR) as the study's design in the title and/or the abstract if that iss a main purpose of the study	1-2
Introduction			
2	Background	Explain the scientific Background and rationale for the reported study. What isthe exposure? Is a potential causal relationship between exposure and outcome plausible? Justify why MR is a helpful method to address the study question.	3
3	Objectives	State specific objectives clearly, including prespecified causal hypotheses (if any). State that MR is a method that, under specific assumptions intends to estimate causal effects.	3
Methods			
4	Study design and data sources	Present key elements ofthe study design early in the article. Consider including a table listing sources of data for all phases of the study. For each data source contributing to the analysis, describe the following	4
	a	Setting: Describe the study design and the underying population, if posible. Describe the setting, locations, and relevant dates, including periods of recruitment, exposure, follow-up, and data collection, when available.	
	b	Participants: Report theelsblity criteria and the soures and methods of selection of participants. Report the sample size and whether any power or sample size calculations were carried out prior to the main analysis.	
	c	Describe measurement, quality control, and selection of genetic variants.	
	d	For each exposure, outcome, and other relevant variables, describe methods of assessment and diagnostic criteria for diseases.	
	e	Provide details of ethics committee approval and participant informed consent, if relevant.	
5	Assumptions	Explicitly state the 3 core instrumental variable (IV) assumptions for the main analysis relevance, independence, and exclusion restriction), as well assumptions for any additional or sensitivity analysis.	4
6	Statistical methods: main analysis	Describe statistical methods and statistics used.	5

	a	Describe how quantitative variables were handled in the analyses (ie, scale, units, model).	
	b	Describe how genetic variants were handled in the analyses and, if applicable, how their weights were selected.	
	c	Describe the MR estimator (eg, 2-stage least squares, Wald ratio) and related statistics. Detail the included covariates and, in case of 2-sample MR, whether the same covariate set was used for adjustment in the 2 samples.	
	d	Explain how missing data were addressed.	
	e	If apliable indicate how multiple testing was addressed.	
7	Assessment of assumptions	Describe any methods or prior knowledge used to assess the assumptions or justify their validity.	5
8	Sensitivity analyses and additional analyses	Describe any sensitivity analyses or additional analyses performed (eg, comparison of effect estimates from different approaches, independent replication, bias analytic techniques, vaigation of instrument, simulations).	6
9	Software and preregistration		6
	a	Name statistical software and package(s), including version and settings used.	
	b	State whether the study protocol and details were preregistered (as well as when and where).	
**Results**			
10	Descriptive data		6
	a	Report the numbers of indviduals at each stage of included studies and reasons for exclusion, Consider use of a low diagram.	
	b	Report summary statistics for phenotypic exposure(s), outcome(s), and other relevant variables (eg, means, SDs, proportions).	
	c	If the data sources include meta-analyses of previous studes, provide the assessments of heterogeneity across these studies.	
	d	For 2-sample MR:<br>i. Provide justification of the simialrity ofthe genetic varant-exposure associations between the exposure and outcome samples.<br>ii. Provide information on the number of individuals who overlap between the exposure and outcome studies.	

11	Main results		7
	a	Report the associations between genetic variant and exposure and between genetic variant and outcome, preferably on an interpretable scale.	
	b	Report MR estimates of the relationship between exposure and outcome and the measures of uncertainty from the MR analysis, on an interpretable scale, sch as odds ratio o relative isk per SD difference.	
	c	If relevant, consider translating estimates of relative risk into absolute risk for a meaningful time period.	
	d	Consider plots to visualize results (eg, forest plot, scatter plot of association between genetic variants and outcome vs. between genetic variants and exposure).	
12	Assessment of assumptions		7
	a	Report the assessment of the validity ofthe assumptions.	
	b	Report any additional statistics (eg, assessments of heterogeneity across genetic variants, such as I^2, Q statistic, or E-value).	
13	Sensitivity analyses and additional analyses		7
	a	Report any sensitivity analyses to assess the robustness of the main results to violations of the assumptions.	
	b	Report results from other sensitivity analyses or addtional analyses.	
	c	Report any assessment of the direction of the causal relationship (eg, bidirectional MR).	
	d	When relevant, report and compare with estimates from non-MR analyses.	
	e	Consider addtional plot to visualize results (eg, leave-one-out analyses).	
**Discussion**			
14	Key results	Summarize key eslts with reference to study objectives.	7
15	Limitations	Discuss limitations of the study, taking into account the validity of the IV assumptions, other sources of potential bias, and imprecision. Dscuss both direction and magnitude of any potential bias and any efforts to address them.	9
16	Interpretation		8

	a	Meaning: Give a cautious overall interpretation of results in the context of their limitations and in comparison with other studies.	
	b	Mechanism: Discuss underlying biological mechanisms that could drive a potential causal relationship between the investigated exposure and the outcome, and whether the gene-environment equivalence assumptions reasonable. Use causal language carefully, clarifying that IV estimates may provide causal effects only under certain assumptions.	
	c	Clinical relevance: Discuss whether the results have clinical or public policy relevance, and to what extent they inform effect sizes of possible interventions.	
17	Generalizabilty	Discuss the generalizability of the study results (a) to ther populations, (b) across other exposure periods/timings, and (c) across other levels of exposure.	10
**Other Information**			
18	Funding	Describe sources of funding and the role of funders inthe present study and, if applicable, sources of funding for the databases and original study or studies on which the present study is based.	10
19	Data and data sharing	Provide the data used to perform all analyses of report where and how the data can be accessed, and reference these sources in the article. Provide the statistical code needed to reproduce the results in the article or report whether the code is publicly accessible and, if so, where.	10
20	Conflicts of interest	All authors should declare all potential conflicts of interest.	10

Table S2. Data sources of the diseases/traits/confounders included in genetic correlation in MR analysis.

Disease or trait	Vaiables	Data source	Ancestry	Sample size	Reference
Fatty liver	Exposure	Million Veteran Program (MVP)	European American, African American, Hispanic American and Asian American	Cases: 90,408<br>Controls: 128,187	[PMID: 37709864]
Systolic blood pressure	Outcome	UK Biobank	European	Cases: 327,302<br>Controls: 408,455	[PMID: 30224653]
Diastolic blood pressure	Outcome				
Pulse pressure	Outcome				
Body mass index (BMI)	Potential confounder	MRC-IEU	European	Cases: 48,286<br>Controls: 250,671	[PMID: 27841877]
Total cholesterol	Potential confounder				
Total triglycerides	Potential confounder				
Type 2 Diabetes (T2DM)	Potential confounder				
Insulin resistance	Potential confounder				
Alcohol consumption	Potential confounder				
Current tobacco smoking	Potential confounder				

Table S3. Genetic instruments for fatty liver

SNP	Nominated Gene	Chr	Position	effect allele	non-effect allele	effect allele frequency	beta.exposure	se.exposure	pval.exposure	beta.outcome	se.outcome	pval.outcome
rs11668950	IFI30;MPV17L2;PIK3R2	19	18,282,940	A	G	0.258	0.106	0.026	4.070E-05	-0.025	0.011	2.616E-02
rs1337101	SLC30A10	1	219,726,100	T	G	0.297	-0.055	0.027	4.400E-02	-0.012	0.010	2.363E-01
rs13389219	COBLL1;SCN2A	2	165,528,876	T	C	0.432	-0.095	0.023	3.860E-05	-0.024	0.010	1.878E-02
rs17036160	PPARG	3	12,329,783	T	C	0.115	-0.082	0.036	2.500E-02	-0.001	0.015	9.317E-01
rs17598226	MTTP	4	100,496,891	G	C	0.261	-0.112	0.026	1.450E-05	-0.038	0.010	2.674E-04
rs1801689	APOH	17	64,210,580	C	A	0.031	0.260	0.065	5.650E-05	0.043	0.036	2.381E-01
rs2207132	MAFB	20	39,142,516	A	G	0.028	0.297	0.088	6.900E-04	0.136	0.035	1.062E-04
rs2642438	MTARC1	1	220,970,028	A	G	0.274	-0.180	0.027	1.500E-11	-0.008	0.011	4.790E-01
rs2943652	MIR5702;lnc-RHBDD1-6;lnc-RHBDD1-3	2	227,108,446	C	T	0.353	-0.062	0.023	6.000E-03	-0.037	0.010	3.357E-04
rs2980888	lnc-TRIB1-2;WASHC5	8	126,507,308	T	C	0.286	0.178	0.025	2.420E-12	0.042	0.011	6.660E-05
rs3935942	CD276	15	73,971,361	A	C	0.384	0.054	0.022	1.500E-02	0.013	0.009	1.518E-01
rs4918722	GPAM	10	113,947,040	C	T	0.277	0.127	0.025	4.180E-07	0.011	0.010	3.113E-01

rs5117	APOE;APOC1	19	45,418,790	C	T	0.231	-0.155	0.029	1.050E-07	0.012	0.013	3.926E-01
rs56094641	FTO	16	53,806,453	G	A	0.377	0.089	0.024	2.390E-04	0.030	0.010	1.947E-03
rs58542926	TM6SF2	19	19,379,549	T	C	0.070	0.532	0.042	8.230E-37	-0.051	0.018	5.204E-03
rs7041363	AKNA	9	117,146,043	G	C	0.459	-0.062	0.023	7.000E-03	0.012	0.009	2.072E-01
rs738408	PNPLA3	22	44,324,730	T	C	0.239	0.686	0.025	9.960E-161	-0.019	0.011	8.135E-02

Abbreviations: Chr, chromosome; SNPs, single nucleotide polymorphisms.

Table S4. List of the 77 cALT SNPs

SNP	Nominated Gene	Chr	Position	effect allele	non-effect allele	effect allele frequency	beta.exposure	se.exposure	pval.exposure	beta.outcome	se.outcome	pval.outcome	Note
rs10201587	CASP8	2	202,202,791	G	A	0.477	-0.044	0.006	4.180E-12	-0.004	0.010	6.878E-01	
rs10433937	HSD17B13	4	88,230,100	G	T	0.255	-0.081	0.008	5.280E-26	0.009	0.011	3.912E-01	
rs1047891	CPS1	2	211,540,507	A	C	0.325	0.037	0.007	2.760E-08	-0.025	0.011	1.847E-02	
rs10774625	SH2B3;CUX2;ATXN2;LINC01405	12	111,910,219	A	G	0.465	0.039	0.007	9.750E-09	0.067	0.011	2.690E-10	
rs10883451	ERLIN1	10	101,924,418	C	T	0.437	-0.152	0.007	2.650E-112	0.000	0.010	9.670E-01	
rs11601507	TRIM5	11	5,701,074	A	C	0.072	0.099	0.013	1.530E-14	0.073	0.018	3.400E-05	
rs11621792	NYNRIN	14	24,871,926	T	C	0.415	0.042	0.007	5.480E-10	0.008	0.011	4.719E-01	
rs11668950	IFI30;MPV17L2;PIK3R2	19	18,282,940	A	G	0.258	0.046	0.007	2.220E-10	-0.025	0.011	2.616E-02	
rs11683367	EFHD1	2	233,510,011	C	T	0.402	-0.057	0.007	6.380E-18	-0.013	0.010	1.999E-01	
rs11683409	MERTK	2	112,770,134	G	C	0.410	0.044	0.007	9.190E-12	-0.005	0.010	6.284E-01	
rs12149380	DHODH;HP	16	72,043,546	G	C	0.228	0.048	0.008	2.250E-09	0.000	0.011	9.941E-01	

rs12500824	SHROOM3	4	77,416,627	A	G	0.391	0.047	0.007	7.920E-13	0.025	0.010	9.962E-03	
rs132665	APOL3	22	36,564,170	G	A	0.145	-0.066	0.010	1.180E-11	0.032	0.014	2.117E-02	
rs1337101	SLC30A10	1	219,726,100	T	G	0.297	-0.052	0.007	1.980E-12	-0.012	0.010	2.363E-01	
rs13389219	COBLL1;SCN2A	2	165,528,876	T	C	0.432	-0.053	0.007	8.200E-16	-0.024	0.010	1.878E-02	
rs146774114	DNAJC22	12	49,743,142	A	G	0.015	-0.157	0.028	2.500E-08	0.049	0.049	3.121E-01	
rs148337160	FBXL15;ELOVL3;PSD	10	104,166,504	C	T	0.063	0.088	0.013	2.890E-11	0.064	0.022	4.063E-03	
rs1547014	CHEK2	22	29,100,711	T	C	0.342	-0.064	0.007	1.570E-21	-0.015	0.010	1.593E-01	
rs1626329	P2RX7	12	121,622,023	T	C	0.388	0.05	0.007	2.610E-14	-0.004	0.010	6.804E-01	
rs1658943	UHRF2	9	6,676,953	T	C	0.158	-0.049	0.009	3.540E-08	-0.006	0.012	6.425E-01	
rs17036160	PPARG	3	12,329,783	T	C	0.115	-0.068	0.011	3.390E-10	-0.001	0.015	9.317E-01	
rs174535	FADS2	11	61,551,356	C	T	0.336	-0.064	0.007	1.590E-20	-0.022	0.010	2.887E-02	SNP associated with impaired VLDL secretion
rs17598226	MTTP	4	100,496,891	G	C	0.261	-0.042	0.007	5.610E-09	-0.038	0.010	2.674E-04	SNP associated with impaired VLDL secretion
rs1801689	APOH	17	64,210,580	C	A	0.031	0.17	0.019	1.460E-18	0.043	0.036	2.381E-01	

rs2207132	MAFB	20	39,142,516	A	G	0.028	0.178	0.028	1.580E-10	0.136	0.035	1.062E-04	removed because of linkage disequilibrium
rs2296285	FLT1	13	29,009,673	A	T	0.335	0.039	0.007	1.760E-08	0.044	0.010	7.330E-06	
rs2642438	MTARC1	1	220,970,028	A	G	0.274	-0.075	0.007	6.650E-24	-0.008	0.011	4.790E-01	
rs2727324	SMARCD2;DDX42	17	61,922,102	C	G	0.368	0.038	0.007	1.240E-08	-0.001	0.010	9.076E-01	
rs2737217	TRPS1	8	116,630,311	A	G	0.392	0.044	0.007	3.920E-10	0.008	0.010	4.327E-01	
rs28929474	SERPINA1	14	94,844,947	T	C	0.017	0.492	0.027	9.010E-73	-0.147	0.045	1.173E-03	removed because of linkage disequilibrium
rs2943652	MIR5702;lnc-RHBDD1-6;lnc-RHBDD1-3	2	227,108,446	C	T	0.353	-0.062	0.007	1.040E-19	-0.037	0.010	3.357E-04	
rs2980888	lnc-TRIB1-2;WASHC5	8	126,507,308	T	C	0.286	0.13	0.007	4.210E-72	0.042	0.011	6.660E-05	
rs340009	RORA	15	60,899,639	A	C	0.399	0.043	0.007	3.650E-11	-0.006	0.009	5.010E-01	
rs34123446	MLXIP	12	122,511,238	G	A	0.444	-0.043	0.007	2.820E-11	-0.005	0.010	6.091E-01	
rs35199395	HKDC1	10	70,983,936	G	C	0.411	-0.039	0.007	2.790E-08	0.002	0.010	8.654E-01	
rs36086195	EPHA2	1	16,510,894	C	T	0.475	-0.043	0.007	4.500E-10	-0.026	0.010	7.179E-03	
rs3810367	STAP2;MPND	19	4,342,847	G	T	0.374	0.037	0.007	3.770E-08	0.012	0.010	2.071E-01	

rs3852142	MAP3K1;lnc-ANKRD55-8	5	55,796,968	T	A	0.077	-0.072	0.013	1.900E-08	-0.037	0.019	5.106E-02	
rs3935942	CD276	15	73,971,361	A	C	0.384	0.055	0.007	2.720E-15	0.013	0.009	1.518E-01	
rs4484649	SOX7;RP1L1;C8orf74	8	10,571,491	C	A	0.419	0.045	0.007	1.380E-11	0.018	0.010	7.004E-02	
rs4683438	PAQR9;U2SURP	3	142,652,559	T	G	0.338	-0.047	0.007	4.730E-12	-0.013	0.011	2.401E-01	
rs4711750	C6orf223;LINC02537;SCIRT	6	43,757,082	A	T	0.465	0.037	0.007	4.790E-08	-0.042	0.010	2.550E-05	removed because palindromic
rs4734654	KLF10	8	103,669,991	G	A	0.342	-0.044	0.007	1.550E-10	-0.011	0.010	2.461E-01	
rs4782568	OSGIN1;MLYCD	16	83,980,529	G	C	0.420	-0.064	0.007	8.820E-21	0.018	0.011	9.246E-02	removed because palindromic
rs4805033	CEBPA;CEBPG	19	33,839,554	G	A	0.439	-0.037	0.007	3.060E-08	-0.014	0.010	1.579E-01	
rs4841132	PPP1R3B	8	9,183,596	A	G	0.106	0.123	0.011	6.620E-32	0.000	0.016	9.933E-01	
rs4918722	GPAM	10	113,947,040	C	T	0.277	0.077	0.008	1.750E-23	0.011	0.010	3.113E-01	
rs5117	APOE;APOC1	19	45,418,790	C	T	0.231	-0.072	0.008	2.210E-20	0.012	0.013	3.926E-01	SNP associated with impaired VLDL secretion
rs56094641	FTO	16	53,806,453	G	A	0.377	0.042	0.007	1.360E-09	0.030	0.010	1.947E-03	
rs56175344	PANX1	11	93,864,393	G	C	0.124	-0.128	0.010	4.600E-40	-0.011	0.015	4.451E-01	

rs58542926	TM6SF2	19	19,379,549	T	C	0.070	0.209	0.013	6.540E-62	-0.051	0.018	5.204E-03	SNP associated with impaired VLDL secretion
rs60315134	PRAG1;lnc-PRAG1-3	8	8,670,599	G	A	0.457	0.036	0.007	2.880E-08	0.014	0.010	1.394E-01	removed because of linkage disequilibrium
rs6059896	ITCH	20	33,111,783	C	T	0.485	0.047	0.006	1.800E-13	0.004	0.009	6.777E-01	
rs61791108	SLC2A2	3	170,732,742	A	G	0.030	0.111	0.019	2.730E-09	-0.052	0.029	7.358E-02	
rs6541349	CCDC18	1	93,787,867	C	T	0.268	0.049	0.008	1.760E-09	0.009	0.011	4.303E-01	
rs6543007	TBC1D8;IL1R2	2	101,663,584	T	C	0.431	0.036	0.007	5.000E-08	0.008	0.010	3.950E-01	
rs6734238	IL1RN	2	113,841,030	G	A	0.407	-0.057	0.006	4.940E-19	-0.023	0.010	2.218E-02	
rs686250	HLA-DRA;HLA-DQA2;HLA-DQB2;HLA-DOB;PSMB8;PSMB9	6	32,585,055	A	G	0.415	-0.039	0.007	3.060E-09	-0.013	0.012	2.762E-01	
rs687621	ABO	9	136,137,065	G	A	0.354	0.043	0.007	7.910E-11	0.042	0.009	7.140E-06	
rs7041363	AKNA	9	117,146,043	G	C	0.459	-0.13	0.007	1.020E-81	0.012	0.009	2.072E-01	removed because palindromic
rs7168849	ANPEP	15	90,346,227	G	A	0.235	-0.064	0.009	1.670E-11	0.005	0.017	7.884E-01	
rs73024760	ABCB11	2	169,885,122	T	C	0.045	0.104	0.016	7.590E-11	0.004	0.024	8.642E-01	
rs738408	PNPLA3	22	44,324,730	T	C	0.239	0.267	0.008	1.000E-200	-0.019	0.011	8.135E-02	SNP associated with impaired VLDL secretion

rs74816838	FCGR2A;FCGR2B	1	161,643,560	T	C	0.106	0.078	0.012	1.510E-10	0.024	0.019	2.252E-01	
rs7599	TMEM147;ATP4A	19	36,038,390	A	G	0.388	0.046	0.007	1.050E-12	0.004	0.010	6.597E-01	
rs7653249	PCCB	3	136,005,792	C	G	0.229	-0.076	0.008	3.510E-22	-0.026	0.011	1.999E-02	
rs79598313	SYTL1;GPN2;PIGV;KDF1;lnc-SFN-1	1	27,284,913	T	C	0.021	0.172	0.024	4.910E-13	0.058	0.035	9.209E-02	
rs799165	MLXIPL;BCL7B	7	73,052,057	A	T	0.125	0.06	0.010	1.320E-09	0.015	0.015	3.045E-01	SNP associated with impaired VLDL secretion
rs8082024	KAT7	17	47,945,460	C	T	0.322	0.042	0.007	2.320E-08	0.005	0.011	6.654E-01	
rs8108722	S1PR2	19	10,347,084	T	C	0.220	0.044	0.008	2.250E-08	0.004	0.012	7.028E-01	
rs848559	CRIM1	2	36,694,497	T	A	0.141	-0.055	0.010	1.580E-08	-0.029	0.014	4.511E-02	
rs934295	TM4SF1	3	149,122,431	A	T	0.360	0.058	0.007	2.930E-16	-0.001	0.010	9.388E-01	
rs9668670	KRT84;KRT82;KRT74	12	53,278,512	T	A	0.346	-0.054	0.007	2.890E-15	0.000	0.010	9.874E-01	
rs574044675	-	3	172,274,232	C	A	0.023							missing in gene-outcome dataset (no alternative SNP present)
rs138033684	-	6	71,895,252	G	T	0.006							missing in gene-outcome dataset (no alternative SNP present)
rs115038698	ABCB4	7	87,024,718	T	C	0.012							missing in gene-outcome dataset (no alternative SNP present)

rs141505249	GPT	8	145,732,114	C	G	0.003	missing in gene-outcome dataset (no alternative SNP present)

Abbreviations: cALT, chronically-elevated serum alanine aminotransferase levels; Chr, chromosome; SNPs, single nucleotide polymorphisms; VLDL, very low-density lipoprotein.

Table S5. List of the 22 imaging-based fatty liver SNPs

SNP	Nominated Gene	Chr	Position	effect allele	non-effect allele	effect allele frequency	beta.exposure	se.exposure	pval.exposure	beta.outcome	se.outcome	pval.outcome	Note
rs11668950	IFI30;MPV17L2;PIK3R2	19	18,282,940	A	G	0.261	0.022	0.005	4.940E-06	-0.025	0.011	2.616E-02	
rs1337101	SLC30A10	1	219,726,100	T	G	0.307	-0.013	0.005	5.000E-03	-0.012	0.010	2.363E-01	
rs13389219	COBLL1;SCN2A	2	165,528,876	T	C	0.399	-0.019	0.005	4.860E-05	-0.024	0.010	1.878E-02	
rs17036160	PPARG	3	12,329,783	T	C	0.112	-0.018	0.005	1.860E-04	-0.001	0.015	9.317E-01	
rs17598226	MTTP	4	100,496,891	G	C	0.257	-0.026	0.005	5.210E-08	-0.038	0.010	2.674E-04	SNP associated with impaired VLDL secretion
rs1801689	APOH	17	64,210,580	C	A	0.029	0.023	0.005	1.240E-06	0.043	0.036	2.381E-01	
rs2207132	MAFB	20	39,142,516	A	G	0.032	0.015	0.005	2.000E-03	0.136	0.035	1.062E-04	removed because of linkage disequilibrium
rs2642438	MTARC1	1	220,970,028	A	G	0.282	-0.033	0.005	2.260E-12	-0.008	0.011	4.790E-01	
rs2737217	TRPS1	8	116,630,311	A	G	0.421	0.009	0.005	4.700E-02	0.008	0.010	4.327E-01	
rs2943652	MIR5702;lnc-RHBDD1-6;lnc-RHBDD1-3	2	227,108,446	C	T	0.351	-0.014	0.005	3.000E-03	-0.037	0.010	3.357E-04	
rs2980888	lnc-TRIB1-2;WASHC5	8	126,507,308	T	C	0.296	0.030	0.005	2.860E-10	0.042	0.011	6.660E-05	

rs34123446	MLXIP	12	122,511,238	G	A	0.467	-0.011	0.005	1.900E-02	-0.005	0.010	6.091E-01	
rs3935942	CD276	15	73,971,361	A	C	0.417	0.011	0.005	2.400E-02	0.013	0.009	1.518E-01	
rs4782568	OSGIN1;MLYCD	16	83,980,529	G	C	0.446	-0.011	0.005	1.900E-02	0.018	0.011	9.246E-02	removed because palindromic
rs4918722	GPAM	10	113,947,040	C	T	0.274	0.038	0.005	1.460E-15	0.011	0.010	3.113E-01	
rs5117	APOE;APOC1	19	45,418,790	C	T	0.224	-0.039	0.005	1.840E-16	0.012	0.013	3.926E-01	SNP associated with impaired VLDL secretion
rs56094641	FTO	16	53,806,453	G	A	0.386	0.013	0.005	7.000E-03	0.030	0.010	1.947E-03	
rs58542926	TM6SF2	19	19,379,549	T	C	0.073	0.106	0.005	3.380E-112	-0.051	0.018	5.204E-03	SNP associated with impaired VLDL secretion
rs687621	ABO	9	136,137,065	G	A	0.321	0.011	0.005	2.700E-02	0.042	0.009	7.140E-06	
rs7041363	AKNA	9	117,146,043	G	C	0.474	-0.011	0.005	2.100E-02	0.012	0.009	2.072E-01	removed because palindromic
rs738408	PNPLA3	22	44,324,730	T	C	0.226	0.125	0.005	5.400E-155	-0.019	0.011	8.135E-02	SNP associated with impaired VLDL secretion
rs7653249	PCCB	3	136,005,792	C	G	0.251	-0.013	0.005	5.000E-03	-0.026	0.011	1.999E-02	

Abbreviations: Chr, chromosome; MASH, non-alcoholic fatty liver disease; SNPs, single nucleotide polymorphisms; VLDL, very low-density lipoprotein.

Table S6. List of the 36 biopsy-confirmed fatty liver SNPs

SNP	Nominated Gene	Chr	Position	effect allele	non-effect allele	effect allele frequency	beta.exposure	se.exposure	pval.exposure	beta.outcome	se.outcome	pval.outcome	Note
rs10433937	HSD17B13	4	88,230,100	G	T	0.259	-0.219	0.029	1.990E-14	0.009	0.011	3.912E-01	
rs10883451	ERLIN1	10	101,924,418	C	T	0.463	-0.125	0.024	1.340E-07	0.000	0.010	9.670E-01	
rs11601507	TRIM5	11	5,701,074	A	C	0.073	0.109	0.051	3.151E-02	0.073	0.018	3.400E-05	
rs11621792	NYNRIN	14	24,871,926	T	C	0.441	0.057	0.026	2.908E-02	0.008	0.011	4.719E-01	
rs11668950	IFI30;MPV17L2;PIK3R2	19	18,282,940	A	G	0.269	0.106	0.026	4.070E-05	-0.025	0.011	2.616E-02	
rs132665	APOL3	22	36,564,170	G	A	0.145	-0.092	0.040	2.174E-02	0.032	0.014	2.117E-02	
rs1337101	SLC30A10	1	219,726,100	T	G	0.317	-0.055	0.027	4.414E-02	-0.012	0.010	2.363E-01	
rs13389219	COBLL1;SCN2A	2	165,528,876	T	C	0.383	-0.095	0.023	3.860E-05	-0.024	0.010	1.878E-02	
rs146774114	DNAJC22	12	49,743,142	A	G	0.015	-0.287	0.129	2.594E-02	0.049	0.049	3.121E-01	
rs17036160	PPARG	3	12,329,783	T	C	0.117	-0.082	0.036	2.508E-02	-0.001	0.015	9.317E-01	
rs17598226	MTTP	4	100,496,891	G	C	0.253	-0.112	0.026	1.450E-05	-0.038	0.010	2.674E-04	SNP associated with impaired VLDL secretion

rs1801689	APOH	17	64,210,580	C	A	0.031	0.260	0.065	5.650E-05	0.043	0.036	2.381E-01	
rs2207132	MAFB	20	39,142,516	A	G	0.033	0.297	0.088	6.900E-04	0.136	0.035	1.062E-04	removed because of linkage disequilibrium
rs2642438	MTARC1	1	220,970,028	A	G	0.278	-0.180	0.027	1.500E-11	-0.008	0.011	4.790E-01	
rs28929474	SERPINA1	14	94,844,947	T	C	0.021	0.543	0.076	7.280E-13	-0.147	0.045	1.173E-03	removed because of linkage disequilibrium
rs2943652	MIR5702;lnc-RHBDD1-6;lnc-RHBDD1-3	2	227,108,446	C	T	0.349	-0.062	0.023	6.033E-03	-0.037	0.010	3.357E-04	
rs2980888	lnc-TRIB1-2;WASHC5	8	126,507,308	T	C	0.312	0.178	0.025	2.420E-12	0.042	0.011	6.660E-05	
rs36086195	EPHA2	1	16,510,894	C	T	0.425	-0.067	0.023	3.698E-03	-0.026	0.010	7.179E-03	
rs3935942	CD276	15	73,971,361	A	C	0.416	0.054	0.022	1.490E-02	0.013	0.009	1.518E-01	
rs4683438	PAQR9;U2SURP	3	142,652,559	T	G	0.321	-0.070	0.026	7.175E-03	-0.013	0.011	2.401E-01	
rs4711750	C6orf223;LINC02537;SCIRT	6	43,757,082	A	T	0.504	0.056	0.025	2.757E-02	0.042	0.010	2.550E-05	removed because palindromic
rs4734654	KLF10	8	103,669,991	G	A	0.354	-0.080	0.024	1.120E-03	-0.011	0.010	2.461E-01	
rs4918722	GPAM	10	113,947,040	C	T	0.299	0.127	0.025	4.180E-07	0.011	0.010	3.113E-01	
rs5117	APOE;APOC1	19	45,418,790	C	T	0.216	-0.155	0.029	1.050E-07	0.012	0.013	3.926E-01	SNP associated with impaired VLDL secretion

rs56094641	FTO	16	53,806,453	G	A	0.417	0.089	0.024	2.390E-04	0.030	0.010	1.947E-03	
rs58542926	TM6SF2	19	19,379,549	T	C	0.081	0.532	0.042	8.230E-37	-0.051	0.018	5.204E-03	SNP associated with impaired VLDL secretion
rs6734238	IL1RN	2	113,841,030	G	A	0.391	-0.105	0.025	2.280E-05	-0.023	0.010	2.218E-02	
rs686250	HLA-DRA;HLA-DQA2;HLA-DQB2;HLA-DOB;PSMB8;PSMB9	6	32,585,055	A	G	0.383	-0.092	0.028	8.790E-04	-0.013	0.012	2.762E-01	
rs7041363	AKNA	9	117,146,043	G	C	0.481	-0.062	0.023	6.685E-03	0.012	0.009	2.072E-01	removed because palindromic
rs73024760	ABCB11	2	169,885,122	T	C	0.034	0.186	0.069	6.619E-03	0.004	0.024	8.642E-01	
rs738408	PNPLA3	22	44,324,730	T	C	0.292	0.686	0.025	9.960E-161	-0.019	0.011	8.135E-02	SNP associated with impaired VLDL secretion
rs74816838	FCGR2A;FCGR2B	1	161,643,560	T	C	0.138	0.122	0.059	3.828E-02	0.024	0.019	2.252E-01	
rs79598313	SYTL1;GPN2;PIGV;KDF1;lnc-SFN-1	1	27,284,913	T	C	0.023	0.194	0.087	2.582E-02	0.058	0.035	9.209E-02	
rs8082024	KAT7	17	47,945,460	C	T	0.227	0.052	0.026	4.413E-02	0.005	0.011	6.654E-01	
rs848559	CRIM1	2	36,694,497	T	A	0.144	-0.113	0.036	1.494E-03	-0.029	0.014	4.511E-02	
rs9668670	KRT84;KRT82;KRT74	12	53,278,512	T	A	0.331	-0.066	0.024	6.681E-03	0.000	0.010	9.874E-01	

Abbreviations: Chr, chromosome; MASH, non-alcoholic fatty liver disease; SNPs, single nucleotide polymorphisms; VLDL, very low-density lipoprotein.

Table S7. Characteristics of index SNPs represented genetically predicted fatty liver in cALT cohort used in outcome-SBP.

No.	SNP*	effect allele	other allele	exposure				outcome-SBP				Steiger filtering				F-Statistic		Note
				beta	se	pval	eaf	beta	se	pval	eaf	rsq_exposure	rsq_outcome	steiger_pval	steiger_direction	R square	F-Statistic	
1	rs10201587	G	A	-0.045	0.007	1.29E-10	0.499	-0.0175	0.03	0.5593	0.5084	2.52E-04	4.63E-07	2.66E-08	TRUE	0.001012496	166.4152673	
2	rs10883451	C	T	-0.161	0.007	4.66E-117	0.477	0.0113	0.0303	0.7082	0.4851	3.21E-03	1.92E-07	2.79E-94	TRUE	0.012933076	2151.370178	Outlier SNP
3	rs112128680	A	G	0.061	0.01	1.06E-09	0.149	-0.1086	0.0423	0.0102101	0.1497	2.27E-04	8.97E-06	1.00E-05	TRUE	0.000943638	155.0870134	
4	rs11601507	A	C	0.088	0.014	3.26E-10	0.073	0.0507	0.0608	0.4045	0.0691	2.41E-04	9.94E-07	1.20E-07	TRUE	0.001048088	172.2714384	
5	rs11621792	T	C	0.042	0.007	1.97E-09	0.453	-0.0373	0.0313	0.2343	0.4454	2.19E-04	2.05E-06	1.11E-06	TRUE	0.000874207	143.6659543	
6	rs1169292	T	C	0.054	0.008	1.48E-11	0.321	0.098	0.0325	0.00254601	0.3127	2.77E-04	1.24E-05	1.49E-06	TRUE	0.001271137	208.9799635	
7	rs12500824	A	G	0.046	0.008	8.92E-09	0.35	0.214	0.0315	1.05E-11	0.3511	2.01E-04	6.27E-05	0.021635295	TRUE	0.00096278	158.2360086	Outlier SNP
8	rs132665	G	A	-0.069	0.01	5.20E-12	0.154	0.0778	0.0417	0.0620798	0.1561	2.90E-04	4.76E-06	5.43E-08	TRUE	0.001240564	203.9474566	
9	rs1337101	T	G	-0.051	0.008	1.83E-10	0.3	-0.1467	0.0324	6.14E-06	0.3106	2.47E-04	2.78E-05	0.000127327	TRUE	0.00109242	179.5660635	Outlier SNP
10	rs13409360	A	G	-0.06	0.008	6.38E-14	0.397	0.0152	0.0309	0.6215	0.396	3.42E-04	3.32E-07	5.27E-11	TRUE	0.001723615	283.4976386	
11	rs149740	A	G	-0.04	0.007	1.97E-0	0.42	-0.151	0.030	7.86E-07	0.419	2.19E-04	3.36E-05	0.000973	TRUE	0.000862	141.7701	Outlier SNP

	6			2		9	6	3	6		3			599		681	553	
12	rs1547014	T	C	-0.064	0.008	1.24E-15	0.302	-0.0568	0.0326	0.0813299	0.3144	3.90E-04	4.12E-06	8.56E-11	TRUE	0.001726841	284.0291035	
13	rs1594320	G	C	0.053	0.008	3.47E-11	0.296	-0.0091	0.0332	0.7838	0.2951	2.67E-04	1.03E-07	4.46E-09	TRUE	0.001170701	192.4486018	
14	rs174535	C	T	-0.061	0.008	2.44E-14	0.337	-0.0495	0.0317	0.1182	0.3477	3.54E-04	3.32E-06	4.80E-10	TRUE	0.001662774	273.4738202	
15	rs17780834	T	A	0.086	0.015	9.85E-09	0.061	0.026	0.0619	0.6743	0.065	2.00E-04	2.43E-07	5.79E-07	TRUE	0.000847271	139.2356269	
16	rs1801689	C	A	0.176	0.021	5.25E-17	0.032	-0.1297	0.0918	0.1575	0.0333	4.28E-04	3.03E-06	6.59E-12	TRUE	0.001919025	315.7001714	Outlier SNP
17	rs2138157	A	C	-0.064	0.008	1.24E-15	0.353	-0.2556	0.0314	3.67E-16	0.357	3.90E-04	9.03E-05	0.000176275	TRUE	0.001870979	307.781261	
18	rs2377957	A	G	-0.053	0.008	3.47E-11	0.329	-0.0025	0.0319	0.9366	0.347	2.67E-04	8.47E-09	2.70E-09	TRUE	0.001240224	203.891461	
19	rs2642438	A	G	-0.079	0.008	5.34E-23	0.295	-0.0611	0.0333	0.0669407	0.2954	5.94E-04	4.69E-06	4.84E-16	TRUE	0.002595944	427.3503945	
20	rs2792751	T	C	0.072	0.008	2.26E-19	0.293	0.1301	0.0336	0.000109401	0.2801	4.93E-04	2.06E-05	9.90E-11	TRUE	0.002147742	353.4074546	Outlier SNP
21	rs28929474	T	C	0.481	0.028	3.84E-66	0.018	-0.5331	0.1082	8.44E-07	0.021	1.79E-03	3.33E-05	5.87E-41	TRUE	0.008179074	1354.037843	Outlier SNP
22	rs2954038	C	A	0.139	0.008	1.28E-67	0.306	0.1771	0.0333	1.03E-07	0.3009	1.84E-03	3.94E-05	8.47E-41	TRUE	0.00820617	1358.560611	
23	rs3810367	G	T	0.045	0.008	1.86E-08	0.376	0.0151	0.0312	0.6285	0.3769	1.93E-04	3.22E-07	1.10E-06	TRUE	0.000950227	156.170953	

24	rs4148824	G	A	-0.057	0.01	1.20E-08	0.185	-0.0824	0.0396	0.0374999	0.1799	1.98E-04	5.98E-06	2.12E-05	TRUE	0.000979736	161.0255068	Outlier SNP
25	rs429358	C	T	-0.094	0.011	1.28E-17	0.139	0.1377	0.0429	0.001322	0.1532	4.45E-04	1.44E-05	2.70E-10	TRUE	0.002114967	348.003069	
26	rs4684847	T	C	-0.072	0.011	5.93E-11	0.123	-0.1444	0.0462	0.00177399	0.1231	2.61E-04	1.34E-05	4.86E-06	TRUE	0.001118407	183.8423704	
27	rs4734654	G	A	-0.052	0.008	8.03E-11	0.362	0.0048	0.0314	0.8772	0.3647	2.57E-04	3.20E-08	6.32E-09	TRUE	0.00124901	205.3376736	
28	rs4782568	G	C	-0.064	0.008	1.24E-15	0.438	0.0785	0.0313	0.01228	0.4547	3.90E-04	9.13E-06	1.14E-09	TRUE	0.00201651	331.7698688	Being palindromic with intermediate allele frequencies
29	rs4841133	A	G	0.13	0.013	1.52E-23	0.088	0.1487	0.0526	0.00466799	0.0922	6.09E-04	1.09E-05	4.79E-15	TRUE	0.002712653	446.6155394	
30	rs4919741	A	G	-0.057	0.008	1.04E-12	0.344	-0.0145	0.0317	0.6467	0.3416	3.09E-04	2.84E-07	4.19E-10	TRUE	0.001466365	241.123322	
31	rs4940689	A	G	0.054	0.01	6.66E-08	0.205	0.0418	0.0371	0.2606	0.2059	1.78E-04	1.72E-06	1.06E-05	TRUE	0.00095047	156.2109283	
32	rs55868793	G	T	0.059	0.008	1.64E-13	0.408	0.0232	0.0304	0.4465	0.421	3.31E-04	7.88E-07	2.22E-10	TRUE	0.001681574	276.5710571	Outlier SNP
33	rs58542926	T	C	0.222	0.014	1.25E-56	0.075	0.2338	0.0579	5.40E-05	0.0752	1.53E-03	2.25E-05	2.74E-36	TRUE	0.006838155	1130.521542	Outlier SNP
34	rs6717858	C	T	-0.051	0.008	1.83E-10	0.4	-0.2205	0.0306	6.21E-13	0.4042	2.47E-04	7.10E-05	0.007464249	TRUE	0.00124848	205.2504247	
35	rs7041363	G	C	-0.135	0.008	6.87E-64	0.491	0.0968	0.0302	0.001335	0.4852	1.73E-03	1.40E-05	8.57E-44	TRUE	0.009109548	1509.492958	Being palindromic with intermediate allele frequencies
36	rs7117339	T	C	-0.13	0.011	3.14E-32	0.12	0.0852	0.0467	0.0680393	0.1187	8.50E-04	4.55E-06	4.31E-23	TRUE	0.00356928	588.1572274	Outlier SNP

37	rs738409	G	C	0.269	0.0086	8.99E-215	0.228	0.0449	0.0363	0.2168	0.2207	5.92E-03	2.08E-06	3.08E-169	TRUE	0.025473388	4291.93294
38	rs7604422	C	A	-0.053	0.008	3.47E-11	0.401	-0.0279	0.0307	0.3633	0.3982	2.67E-04	1.13E-06	2.16E-08	TRUE	0.001349438	221.8703698
39	rs79598313	T	C	0.18	0.025	6.02E-13	0.023	0.6651	0.0996	2.40E-11	0.0252	3.16E-04	6.27E-05	0.00032148	TRUE	0.001456121	239.4364031
40	rs848559	T	A	-0.061	0.011	2.93E-08	0.151	-0.0747	0.0428	0.0804693	0.1484	1.87E-04	4.23E-06	2.12E-05	TRUE	0.000954057	156.8009793
41	rs9867368	A	G	-0.078	0.009	4.45E-18	0.198	-0.1165	0.0379	0.00211198	0.199	4.57E-04	1.29E-05	7.34E-11	TRUE	0.00193223	317.8766709

Abbreviations: MASH=Non-alcoholic fatty liver disease, SBP=systolic blood pressure, SNP = single nucleotide polymorphism, eaf = effect allele frequency, se = standard error, pval = P value, rsq=r square

Table S8. Characteristics of index SNPs represented genetically predicted fatty liver in both imaging and biopsy cohorts used in outcome-SBP.

No.	SNP*	effect allele	other allele	external replication		exposure-fatty liver				outcome-SBP				Steiger filtering				F-Statistic		Note
				biopsy cohort pval	imaging cohort pval	beta	se	pval	eaf	beta	se	pval	eaf	rsq_exposure	rsq_outcome	steiger_pval	steiger_direction	R square	F-Statistic	
1	rs13389219	T	C	0.00003856	0.00004855	-0.05	0.007	9.14E-13	0.391	-0.2291	0.0308	9.70E-14	0.3946	3.11E-04	7.56E-05	1.07E-03	TRUE	0.001190595	195.7227726	Outlier SNP
2	rs17598226	G	C	0.00001446	5.214E-08	-0.041	0.008	2.98E-07	0.261	-0.0598	0.0348	8.58E-02	0.254	1.60E-04	4.07E-06	1.00E-04	TRUE	0.000648459	106.5428467	
3	rs1801689	C	A	0.00005645	0.000001243	0.176	0.021	5.25E-17	0.032	-0.1297	0.0918	1.58E-01	0.0333	4.28E-04	3.03E-06	6.59E-12	TRUE	0.001919025	315.7001714	Outlier SNP
4	rs2642438	A	G	1.502E-11	2.258E-12	-0.079	0.008	5.34E-23	0.295	-0.0611	0.0333	6.69E-02	0.2954	5.94E-04	4.69E-06	4.84E-16	TRUE	0.002595944	427.3503945	
5	rs2980888	T	C	2.417E-12	2.86E-10	0.139	0.008	1.28E-67	0.306	0.1785	0.0333	8.17E-08	0.3007	1.84E-03	4.00E-05	1.05E-40	TRUE	0.00820617	1358.560611	Outlier SNP
6	rs4918722	C	T	4.184E-07	1.462E-15	0.075	0.008	6.92E-21	0.29	0.1268	0.0336	1.59E-04	0.2806	5.35E-04	1.95E-05	7.05E-12	TRUE	0.002316375	381.2202422	
7	rs5117	C	T	1.053E-07	1.844E-16	-0.08	0.009	6.17E-19	0.222	-0.0837	0.037	2.36E-02	0.2308	4.81E-04	7.36E-06	2.46161E-12	TRUE	0.002210765	363.8008043	
8	rs58542926	T	C	8.227E-37	3.38E-112	0.222	0.014	1.25E-56	0.075	0.2338	0.0579	5.40E-05	0.0752	1.53E-03	2.25E-05	2.74E-36	TRUE	0.006838155	1130.521542	
9	rs738408	T	C	9.96E-161	5.4E-155	0.269	0.009	2.74E-196	0.228	0.0446	0.0363	2.19E-01	0.2207	5.41E-03	2.05E-06	1.704E-154	TRUE	0.025473388	4291.93294	Outlier SNP

Abbreviations: SBP=systolic blood pressure, SNP = single nucleotide polymorphism, eaf = effect allele frequency, se = standard error, pval = P value, rsq=r square

Table S9. Characteristics of index SNPs represented genetically predicted fatty liver in cALT cohort used in outcome-DBP.

No.	SNP*	effect allele	other allele	exposure				outcome-DBP				Steiger filtering				F-Statistic		Note
				beta	se	pval	eaf	beta	se	pval	eaf	rsq_exposure	rsq_outcome	steiger_pval	steiger_direction	R square	F-Statistic	
1	rs10201587	G	A	-0.045	0.007	1.29E-10	0.499	-0.0306	0.0172	0.0755997	0.5084	0.000251628	4.20E-06	3.91E-07	TRUE	0.001012496	166.4152673	
2	rs10883451	C	T	-0.161	0.007	4.66E-117	0.477	0.0126	0.0174	0.4672	0.4865	0.003211432	7.04E-07	2.08E-93	TRUE	0.012933076	2151.370178	Outlier SNP
3	rs112128680	A	G	0.061	0.01	1.06E-09	0.149	0.048	0.0245	0.0496204	0.1496	0.000226569	5.14E-06	2.73E-06	TRUE	0.000943638	155.0870134	
4	rs11601507	A	C	0.088	0.014	3.26E-10	0.073	-0.0037	0.0347	0.914	0.0693	0.000240572	1.58E-08	1.86E-08	TRUE	0.001048088	172.2714384	
5	rs11621792	T	C	0.042	0.007	1.97E-09	0.453	-0.0381	0.0181	0.0346298	0.446	0.000219203	6.31E-06	7.28E-06	TRUE	0.000874207	143.6659543	
6	rs1169292	T	C	0.054	0.008	1.48E-11	0.321	0.0946	0.0188	4.67E-07	0.3123	0.000277413	3.39E-05	7.08E-05	TRUE	0.001271137	208.9799635	Outlier SNP
7	rs12500824	A	G	0.046	0.008	8.92E-09	0.35	0.0989	0.0181	4.35E-08	0.3516	0.000201321	3.95E-05	0.003711802	TRUE	0.00096278	158.2360086	Outlier SNP
8	rs132665	G	A	-0.069	0.01	5.20E-12	0.154	0.0757	0.0241	0.00168	0.1564	0.000289876	1.33E-05	9.19E-07	TRUE	0.001240564	203.9474566	Outlier SNP
9	rs1337101	T	G	-0.051	0.008	1.83E-10	0.3	0.0276	0.0186	0.1369	0.3118	0.000247453	2.91E-06	2.59E-07	TRUE	0.00109242	179.5660635	
10	rs13409360	A	G	-0.06	0.008	6.38E-14	0.397	-0.0106	0.0177	0.550399	0.3955	0.000342463	4.80E-07	6.31E-11	TRUE	0.001723615	283.4976386	
11	rs149740	A	G	-0.0	0.00	1.97E-0	0.42	-0.0855	0.01	1.11E-06	0.41	0.0002192	3.16E-05	0.000750	TRUE	0.000862	141.7701	Outlier SNP

	6			42	7	9	6		76		92	03		624		681	553	
12	rs1547014	T	C	-0.064	0.008	1.24E-15	0.302	-0.029	0.0188	0.1238	0.3159	0.000389629	3.18E-06	4.39E-11	TRUE	0.001726841	284.0291035	
13	rs1594320	G	C	0.053	0.008	3.47E-11	0.296	-0.0046	0.019	0.8078	0.2955	0.000267236	7.85E-08	3.74E-09	TRUE	0.001170701	192.4486018	
14	rs174535	C	T	-0.061	0.008	2.44E-14	0.337	0.0575	0.0182	0.001557	0.3483	0.00035397	1.32E-05	2.50E-08	TRUE	0.001662774	273.4738202	Outlier SNP
15	rs17780834	T	A	0.086	0.015	9.85E-09	0.061	-0.0995	0.0354	0.00498299	0.0652	0.000200155	1.06E-05	6.41E-05	TRUE	0.000847271	139.2356269	
16	rs1801689	C	A	0.176	0.021	5.25E-17	0.032	-0.0046	0.053	0.9303	0.0331	0.000427603	1.13E-08	8.10E-14	TRUE	0.001919025	315.7001714	Outlier SNP
17	rs2138157	A	C	-0.064	0.008	1.24E-15	0.353	-0.1356	0.018	5.27E-14	0.3563	0.000389629	7.53E-05	4.88E-05	TRUE	0.001870979	307.781261	
18	rs2377957	A	G	-0.053	0.008	3.47E-11	0.329	0.0328	0.0184	0.0748307	0.3475	0.000267236	4.31E-06	1.69E-07	TRUE	0.001240224	203.891461	
19	rs2642438	A	G	-0.079	0.008	5.34E-23	0.295	0.0028	0.0191	0.8834	0.2956	0.000593549	2.92E-08	7.54E-19	TRUE	0.002595944	427.3503945	
20	rs2792751	T	C	0.072	0.008	2.26E-19	0.293	0.073	0.0193	0.0001591	0.2788	0.000493073	1.91E-05	6.00E-11	TRUE	0.002147742	353.4074546	Outlier SNP
21	rs28929474	T	C	0.481	0.028	3.84E-66	0.018	-0.2423	0.0626	0.000107599	0.0211	0.001794049	2.02E-05	7.55E-44	TRUE	0.008179074	1354.037843	
22	rs2954038	C	A	0.139	0.008	1.28E-67	0.306	0.0952	0.0191	6.11E-07	0.3009	0.001835236	3.37E-05	5.41E-42	TRUE	0.00820617	1358.560611	
23	rs3810367	G	T	0.045	0.008	1.86E-08	0.376	-8.00E-04	0.018	0.9667	0.3768	0.000192664	2.67E-09	3.99E-07	TRUE	0.000950227	156.170953	

24	rs4148824	G	A	-0.057	0.01	1.20E-08	0.185	-0.038	0.0227	0.0945105	0.1805	0.000197835	3.77E-06	8.73E-06	TRUE	0.000979736	161.0255068	
25	rs429358	C	T	-0.094	0.011	1.28E-17	0.139	-0.0405	0.0247	0.1014	0.1535	0.000444547	3.73E-06	2.45E-12	TRUE	0.002114967	348.003069	
26	rs4684847	T	C	-0.072	0.011	5.93E-11	0.123	0.011	0.0265	0.6771	0.123	0.000260859	2.31E-07	8.93E-09	TRUE	0.001118407	183.8423704	
27	rs4734654	G	A	-0.052	0.008	8.03E-11	0.362	-0.0168	0.018	0.3507	0.3655	0.00025725	1.16E-06	3.98E-08	TRUE	0.00124901	205.3376736	
28	rs4782568	G	C	-0.064	0.008	1.24E-15	0.438	0.0256	0.018	0.1553	0.4544	0.000389629	2.89E-06	4.72E-11	TRUE	0.00201651	331.7698688	Being palindromic with intermediate allele frequencies
29	rs4841133	A	G	0.13	0.013	1.52E-23	0.088	0.124	0.0301	3.91E-05	0.0922	0.000608661	2.25E-05	2.48E-13	TRUE	0.002712653	446.6155394	
30	rs4919741	A	G	-0.057	0.008	1.04E-12	0.344	-0.024	0.0183	0.1905	0.3417	0.000309083	2.30E-06	3.75E-09	TRUE	0.001466365	241.123322	
31	rs4940689	A	G	0.054	0.01	6.66E-08	0.205	0.0292	0.0215	0.1737	0.2056	0.000177562	2.46E-06	1.59E-05	TRUE	0.00095047	156.2109283	Outlier SNP
32	rs55868793	G	T	0.059	0.008	1.64E-13	0.408	0.0152	0.0176	0.3882	0.4218	0.000331147	9.94E-07	2.72E-10	TRUE	0.001681574	276.5710571	Outlier SNP
33	rs58542926	T	C	0.222	0.014	1.25E-56	0.075	0.2438	0.0334	2.66E-13	0.0751	0.001529063	7.22E-05	3.17E-29	TRUE	0.006838155	1130.521542	
34	rs6717858	C	T	-0.051	0.008	1.83E-10	0.4	-0.1255	0.0176	9.79E-13	0.4038	0.000247453	6.77E-05	0.005902126	TRUE	0.00124848	205.2504247	
35	rs7041363	G	C	-0.135	0.008	6.87E-64	0.491	0.1179	0.0174	1.31E-11	0.485	0.001731311	6.16E-05	2.90E-35	TRUE	0.009109548	1509.492958	Being palindromic with intermediate allele frequencies
36	rs7117339	T	C	-0.13	0.011	3.14E-32	0.12	0.0075	0.0268	0.780899	0.1184	0.000849908	1.04E-07	3.52E-26	TRUE	0.00356928	588.1572274	Outlier SNP

37	rs738409	G	C	0.269	0.0086	8.99E-215	0.228	0.0825	0.021	8.50E-05	0.22	0.005923353	2.06E-05	3.06E-156	TRUE	0.025473388	4291.93294
38	rs7604422	C	A	-0.053	0.008	3.47E-11	0.401	-0.0262	0.0176	0.1371	0.3978	0.000267236	2.95E-06	7.80E-08	TRUE	0.001349438	221.8703698
39	rs79598313	T	C	0.18	0.025	6.02E-13	0.023	0.3286	0.057	8.12E-09	0.0251	0.000315623	4.55E-05	5.44E-05	TRUE	0.001456121	239.4364031
40	rs848559	T	A	-0.061	0.011	2.93E-08	0.151	0.0408	0.0245	0.0962897	0.1482	0.000187255	3.75E-06	1.66E-05	TRUE	0.000954057	156.8009793
41	rs9867368	A	G	-0.078	0.009	4.45E-18	0.198	-0.0361	0.0217	0.0969103	0.1992	0.000457241	3.69E-06	9.07E-13	TRUE	0.00193223	317.8766709

Abbreviations: MASH=Non-alcoholic fatty liver disease, DBP=diastolic blood pressure, SNP = single nucleotide polymorphism, eaf = effect allele frequency, se = standard error, pval = P value, rsq=r square

Table S10. Characteristics of index SNPs represented genetically predicted fatty liver in both imaging and biopsy cohorts used in outcome-DBP.

SNP*	effect allele	other allele	external replication		exposure-MASH				outcome-DBP				Steiger filtering				F-Statistic		Note
			biopsy cohort pval	imaging cohort pval	beta	se	pval	eaf	beta	se	pval	eaf	rsq_exposure	rsq_outcome	steiger_pval	steiger_direction	R square	F-Statistic	
rs13389219	T	C	0.00003856	0.00004855	-0.05	0.007	9.14E-13	0.391	-0.1333	0.0177	4.72E-14	0.3943	3.11E-04	7.55E-05	1.04E-03	TRUE	0.001190595	195.7227726	Outlier SNP
rs17598226	G	C	0.00001446	5.214E-08	-0.041	0.008	2.98E-07	0.261	-0.0249	0.02	2.12E-01	0.2537	1.60E-04	2.08E-06	3.96E-05	TRUE	0.000648459	106.5428467	
rs1801689	C	A	0.00005645	0.000001243	0.176	0.021	5.25E-17	0.032	-0.0046	0.053	9.30E-01	0.0331	4.28E-04	1.13E-08	8.10E-14	TRUE	0.001919025	315.7001714	
rs2642438	A	G	1.502E-11	2.258E-12	-0.079	0.008	5.34E-23	0.295	0.0028	0.0191	8.83E-01	0.2956	5.94E-04	2.92E-08	7.54E-19	TRUE	0.002595944	427.3503945	
rs2980888	T	C	2.417E-12	2.86E-10	0.139	0.008	1.28E-67	0.306	0.0956	0.0191	5.54E-07	0.3007	1.84E-03	3.40E-05	5.96E-42	TRUE	0.00820617	1358.560611	
rs4918722	C	T	4.184E-07	1.462E-15	0.075	0.008	6.92E-21	0.29	0.0729	0.0193	1.59E-04	0.2793	5.35E-04	1.90E-05	5.53E-12	TRUE	0.002316375	381.2202422	
rs5117	C	T	1.053E-07	1.844E-16	-0.08	0.009	6.17E-19	0.222	-0.0559	0.0213	8.70E-03	0.2313	4.81E-04	9.79E-06	6.78626E-12	TRUE	0.002210765	363.8008043	
rs58542926	T	C	8.227E-37	3.38E-112	0.222	0.014	1.25E-56	0.075	0.2438	0.0334	2.66E-13	0.0751	1.53E-03	7.22E-05	3.17E-29	TRUE	0.006838155	1130.521542	Outlier SNP
rs738408	T	C	9.96E-161	5.4E-155	0.269	0.009	2.74E-196	0.228	0.0829	0.021	7.86E-05	0.2199	5.41E-03	2.08E-05	6.031E-142	TRUE	0.025473388	4291.93294	Outlier SNP

Abbreviations: MASH=Non-alcoholic fatty liver disease, DBP=diastolic blood pressure, SNP = single nucleotide polymorphism, eaf = effect allele frequency, se = standard error, pval = P value, rsq=r square

Table S11. Characteristics of index SNPs represented genetically predicted fatty liver in cALT cohort used in outcome-PP.

No.	SNP*	effect allele	other allele	exposure				outcome-PP				Steiger filtering				F-Statistic		Note
				beta	se	pval	eaf	beta	se	pval	eaf	rsq_exposure	rsq_outcome	steiger_pval	steiger_direction	R square	F-Statistic	
1	rs10201587	G	A	-0.045	0.007	1.29E-10	0.499	0.0068	0.0204	0.739	0.5083	0.000251628	1.51E-07	1.41E-08	TRUE	0.001012496	166.4152673	
2	rs10883451	C	T	-0.161	0.007	4.66E-117	0.477	-0.0027	0.0205	0.8973	0.4861	0.003211432	2.38E-08	2.69E-95	TRUE	0.012933076	2151.370178	
3	rs112128680	A	G	0.061	0.01	1.06E-09	0.149	-0.1537	0.0288	9.75E-08	0.1495	0.000226569	3.82E-05	0.001133141	TRUE	0.000943638	155.0870134	Outlier SNP
4	rs11601507	A	C	0.088	0.014	3.26E-10	0.073	0.0528	0.0413	0.2009	0.069	0.000240572	2.21E-06	2.75E-07	TRUE	0.001048088	172.2714384	
5	rs11621792	T	C	0.042	0.007	1.97E-09	0.453	0	0.0213	1	0.4461	0.000219203	0	5.79E-08	TRUE	0.000874207	143.6659543	
6	rs1169292	T	C	0.054	0.008	1.48E-11	0.321	0.0072	0.0221	0.7463	0.3127	0.000277413	1.43E-07	2.38E-09	TRUE	0.001271137	208.9799635	
7	rs12500824	A	G	0.046	0.008	8.92E-09	0.35	0.1161	0.0214	5.52E-08	0.3512	0.000201321	3.99E-05	0.003899106	TRUE	0.00096278	158.2360086	Outlier SNP
8	rs132665	G	A	-0.069	0.01	5.20E-12	0.154	0.0179	0.0284	0.5272	0.1563	0.000289876	5.33E-07	2.27E-09	TRUE	0.001240564	203.9474566	
9	rs1337101	T	G	-0.051	0.008	1.83E-10	0.3	-0.1705	0.022	9.88E-15	0.3105	0.000247453	8.14E-05	0.013901884	TRUE	0.00109242	179.5660635	Outlier SNP
10	rs13409360	A	G	-0.06	0.008	6.38E-14	0.397	0.032	0.021	0.127	0.3962	0.000342463	3.15E-06	8.62E-10	TRUE	0.001723615	283.4976386	
11	rs149740	A	G	-0.04	0.00	1.97E-0	0.42	-0.0702	0.02	0.00073	0.41	0.0002192	1.55E-05	6.74E-05	TRUE	0.000862	141.7701	

	6			2	7	9	6		08	8	96	03					681	553	
12	rs1547014	T	C	-0.064	0.008	1.24E-15	0.302	-0.0275	0.0222	0.2146	0.3147	0.000389629	2.07E-06	1.93E-11	TRUE		0.001726841	284.0291035	
13	rs1594320	G	C	0.053	0.008	3.47E-11	0.296	0.0022	0.0225	0.9236	0.2952	0.000267236	1.30E-08	2.68E-09	TRUE		0.001170701	192.4486018	
14	rs168144	C	T	-0.052	0.007	1.10E-13	0.433	-0.0643	0.0206	0.001821	0.4311	0.000335973	1.31E-05	6.71E-08	TRUE		0.001327723	218.295394	
15	rs174535	C	T	-0.061	0.008	2.44E-14	0.337	-0.1095	0.0215	3.58E-07	0.3472	0.00035397	3.51E-05	2.32E-06	TRUE		0.001662774	273.4738202	Outlier SNP
16	rs17780834	T	A	0.086	0.015	9.85E-09	0.061	0.1269	0.0419	0.002486	0.0651	0.000200155	1.25E-05	0.000101005	TRUE		0.000847271	139.2356269	
17	rs1801689	C	A	0.176	0.021	5.25E-17	0.032	-0.1131	0.0624	0.06964	0.033	0.000427603	4.57E-06	1.21E-11	TRUE		0.001919025	315.7001714	
18	rs2138157	A	C	-0.064	0.008	1.24E-15	0.353	-0.1197	0.0213	1.82E-08	0.3568	0.000389629	4.28E-05	1.33E-06	TRUE		0.001870979	307.781261	Outlier SNP
19	rs2377957	A	G	-0.053	0.008	3.47E-11	0.329	-0.0306	0.0217	0.1596	0.3473	0.000267236	2.67E-06	6.77E-08	TRUE		0.001240224	203.891461	
20	rs2642438	A	G	-0.079	0.008	5.34E-23	0.295	-0.0487	0.0226	0.03135	0.2954	0.000593549	6.31E-06	1.17E-15	TRUE		0.002595944	427.3503945	
21	rs2792751	T	C	0.072	0.008	2.26E-19	0.293	0.0597	0.0228	0.008956	0.2802	0.000493073	9.39E-06	2.40E-12	TRUE		0.002147742	353.4074546	
22	rs28929474	T	C	0.481	0.028	3.84E-66	0.018	-0.2838	0.0737	0.0001182	0.0208	0.001794049	1.99E-05	5.91E-44	TRUE		0.008179074	1354.037843	Outlier SNP
23	rs2954038	C	A	0.139	0.008	1.28E-67	0.306	0.0804	0.0226	0.0003696	0.3012	0.001835236	1.72E-05	1.11E-45	TRUE		0.00820617	1358.560611	

24	rs3810367	G	T	0.045	0.008	1.86E-08	0.376	0.0168	0.0213	0.4302	0.3765	0.000192664	8.46E-07	2.05E-06	TRUE	0.000950227	156.170953	
25	rs4148824	G	A	-0.057	0.01	1.20E-08	0.185	-0.039	0.0269	0.1467	0.1797	0.000197835	2.88E-06	5.94E-06	TRUE	0.000979736	161.0255068	
26	rs429358	C	T	-0.094	0.011	1.28E-17	0.139	0.1879	0.0292	1.28E-10	0.1531	0.000444547	5.60E-05	6.15E-07	TRUE	0.002114967	348.003069	Outlier SNP
27	rs4684847	T	C	-0.072	0.011	5.93E-11	0.123	-0.155	0.0313	7.63E-07	0.1228	0.000260859	3.36E-05	0.000150796	TRUE	0.001118407	183.8423704	Outlier SNP
28	rs4734654	G	A	-0.052	0.008	8.03E-11	0.362	0.032	0.0213	0.1333	0.3649	0.00025725	3.06E-06	1.62E-07	TRUE	0.00124901	205.3376736	
29	rs4782568	G	C	-0.064	0.008	1.24E-15	0.438	0.0521	0.0214	0.01473	0.454	0.000389629	8.02E-06	5.72E-10	TRUE	0.00201651	331.7698688	Being palindromic with intermediate allele frequencies
30	rs4841133	A	G	0.13	0.013	1.52E-23	0.088	0.0346	0.0357	0.3326	0.092	0.000608661	1.27E-06	6.21E-18	TRUE	0.002712653	446.6155394	
31	rs4919741	A	G	-0.057	0.008	1.04E-12	0.344	0.0056	0.0216	0.7962	0.3415	0.000309083	9.11E-08	2.41E-10	TRUE	0.001466365	241.123322	
32	rs4940689	A	G	0.054	0.01	6.66E-08	0.205	0.0106	0.0253	0.6745	0.2059	0.000177562	2.36E-07	2.48E-06	TRUE	0.00095047	156.2109283	
33	rs55868793	G	T	0.059	0.008	1.64E-13	0.408	0.0118	0.0208	0.5694	0.4211	0.000331147	4.32E-07	1.24E-10	TRUE	0.001681574	276.5710571	
34	rs58542926	T	C	0.222	0.014	1.25E-56	0.075	-0.0116	0.0394	0.7683	0.0753	0.001529063	1.17E-07	7.29E-46	TRUE	0.006838155	1130.521542	Outlier SNP
35	rs6717858	C	T	-0.051	0.008	1.83E-10	0.4	-0.0995	0.0208	1.73E-06	0.4042	0.000247453	3.11E-05	0.000198195	TRUE	0.00124848	205.2504247	
36	rs7041363	G	C	-0.135	0.008	6.87E-64	0.491	-0.0162	0.0205	0.4311	0.4856	0.001731311	8.37E-07	1.90E-50	TRUE	0.009109548	1509.492958	Being palindromic with intermediate allele frequencies

37	rs7117339	T	C	-0.13	0.011	3.14E-32	0.12	0.0667	0.0318	0.03572	0.1186	0.000849908	5.96E-06	1.21E-22	TRUE	0.00356928	588.1572274	
38	rs738409	G	C	0.269	0.0086	8.99E-215	0.228	-0.038	0.0247	0.1244	0.2207	0.005923353	3.18E-06	4.71E-168	TRUE	0.025473388	4291.93294	Outlier SNP
39	rs7604422	C	A	-0.053	0.008	3.47E-11	0.401	-5.00E-04	0.0209	0.9807	0.398	0.000267236	7.75E-10	2.21E-09	TRUE	0.001349438	221.8703698	
40	rs79598313	T	C	0.18	0.025	6.02E-13	0.023	0.3207	0.0677	2.17E-06	0.0251	0.000315623	3.04E-05	7.16E-06	TRUE	0.001456121	239.4364031	
41	rs848559	T	A	-0.061	0.011	2.93E-08	0.151	-0.0983	0.029	0.0006946	0.1486	0.000187255	1.56E-05	0.000357624	TRUE	0.000954057	156.8009793	
42	rs9867368	A	G	-0.078	0.009	4.45E-18	0.198	-0.0762	0.0257	0.003007	0.1995	0.000457241	1.19E-05	4.92E-11	TRUE	0.00193223	317.8766709	

Abbreviations: MASH=Non-alcoholic fatty liver disease, DBP=diastolic blood pressure, SNP = single nucleotide polymorphism, eaf = effect allele frequency, se = standard error, pval = P value, rsq=r square

Table S12. Characteristics of index SNPs represented genetically predicted fatty liver in both imaging and biopsy cohorts used in outcome-PP.

No.	SNP*	effect allele	other allele	external replication		exposure-MASH				outcome-PP				Steiger filtering				F-Statistic		Note
				biopsy cohort pval	imaging cohort pval	beta	se	pval	eaf	beta	se	pval	eaf	rsq_exposure	rsq_outcome	steiger_pval	steiger_direction	R square	F-Statistic	
1	rs13389219	T	C	0.00003856	0.00004855	-0.05	0.007	9.14E-13	0.391	-0.0996	0.0209	1.85E-06	0.3947	3.11E-04	3.09E-05	9.72E-06	TRUE	0.001190595	195.7227726	Outlier SNP
2	rs17598226	G	C	0.00001446	5.214E-08	-0.041	0.008	2.98E-07	0.261	-0.0369	0.0236	1.18E-01	0.2545	1.60E-04	3.36E-06	7.57E-05	TRUE	0.000648459	106.5428467	
3	rs1801689	C	A	0.00005645	0.000001243	0.176	0.021	5.25E-17	0.032	-0.1131	0.0624	6.96E-02	0.033	4.28E-04	4.57E-06	1.21E-11	TRUE	0.001919025	315.7001714	
4	rs2642438	A	G	1.502E-11	2.258E-12	-0.079	0.008	5.34E-23	0.295	-0.0487	0.0226	3.14E-02	0.2954	5.94E-04	6.31E-06	1.17E-15	TRUE	0.002595944	427.3503945	
5	rs2980888	T	C	2.417E-12	2.86E-10	0.139	0.008	1.28E-67	0.306	0.0809	0.0226	3.44E-04	0.301	1.84E-03	1.74E-05	1.22E-45	TRUE	0.00820617	1358.560611	Outlier SNP
6	rs4918722	C	T	4.184E-07	1.462E-15	0.075	0.008	6.92E-21	0.29	0.0559	0.0228	1.42E-02	0.2807	5.35E-04	8.14E-06	1.06E-13	TRUE	0.002316375	381.2202422	
7	rs5117	C	T	1.053E-07	1.844E-16	-0.08	0.009	6.17E-19	0.222	-0.027	0.0252	2.85E-01	0.2311	4.81E-04	1.55E-06	3.3652E-14	TRUE	0.002210765	363.8008043	
8	rs58542926	T	C	8.227E-37	3.38E-112	0.222	0.014	1.25E-56	0.075	-0.0116	0.0394	7.68E-01	0.0753	1.53E-03	1.17E-07	7.29E-46	TRUE	0.006838155	1130.521542	
9	rs738408	T	C	9.96E-161	5.4E-155	0.269	0.009	2.74E-196	0.228	-0.038	0.0247	1.24E-01	0.2207	5.41E-03	3.18E-06	2.5619E-153	TRUE	0.025473388	4291.93294	Outlier SNP

Abbreviations: MASH=Non-alcoholic fatty liver disease, DBP=diastolic blood pressure, SNP = single nucleotide polymorphism, eaf = effect allele frequency, se = standard error, pval = P value, rsq=r square

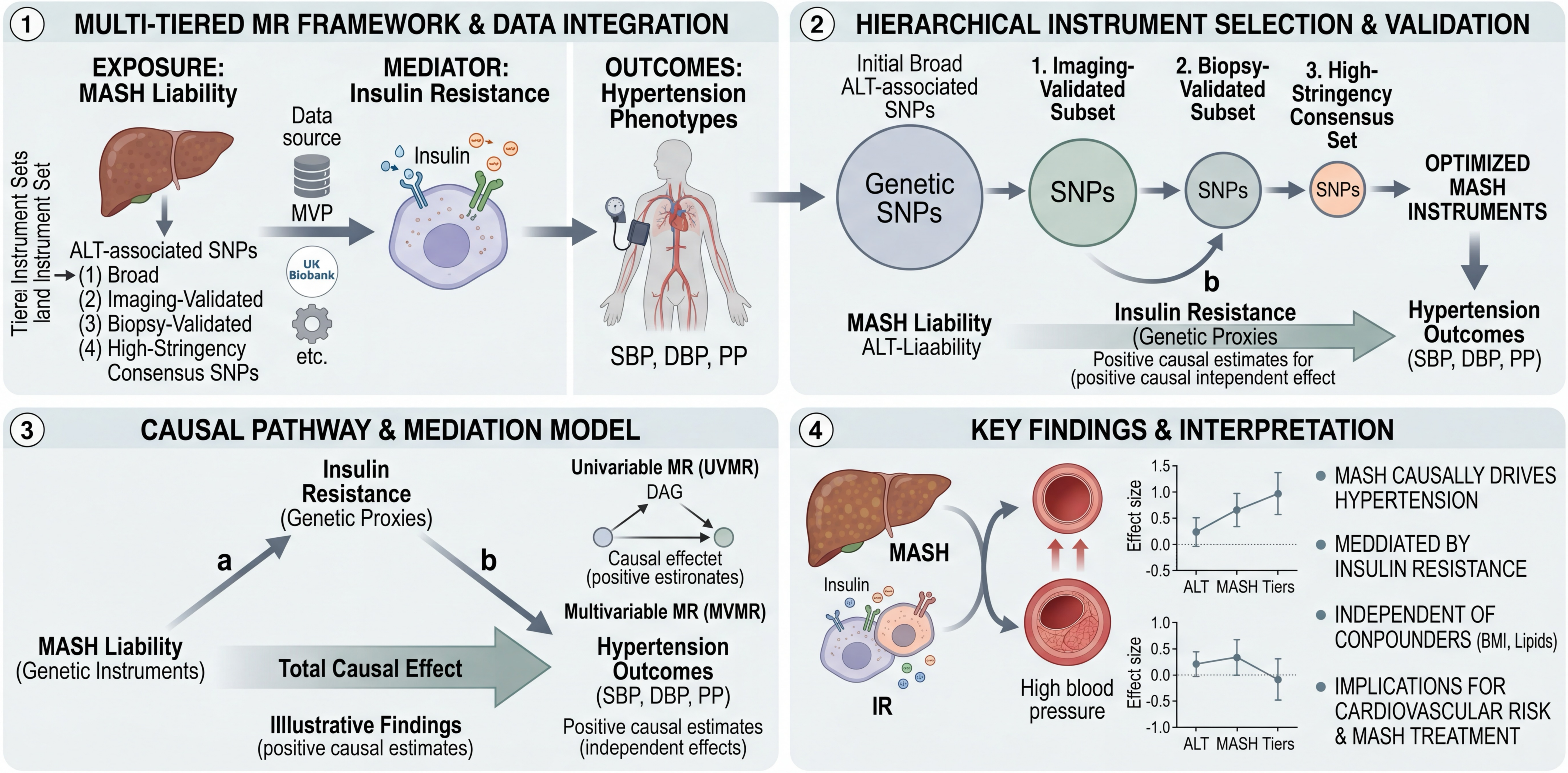
1 MULTI-TIERED MR FRAMEWORK & DATA INTEGRATION
EXPOSURE: MASH Liability
MEDIATOR: Insulin Resistance
OUTCOMES: Hypertension Phenotypes
Tierei Instrument Sets
land Instrument Set
ALT-associated SNPs
(1) Broad
(2) Imaging-Validated
(3) Biopsy-Validated
(4) High-Stringency Consensus SNPs
Data source
MVP
UK Biobank
etc.
Insulin
SBP, DBP, PP
2 HIERARCHICAL INSTRUMENT SELECTION & VALIDATION
Initial Broad ALT-associated SNPs
Genetic SNPs
1. Imaging-Validated Subset
SNPs
2. Biopsy-Validated Subset
SNPs
3. High-Stringency Consensus Set
SNPs
OPTIMIZED MASH INSTRUMENTS
b
MASH Liability
ALT-Liaability
Insulin Resistance
(Genetic Proxies
Positive causal estimates for
(positive causal intependent effect
Hypertension Outcomes
(SBP, DBP, PP)
3 CAUSAL PATHWAY & MEDIATION MODEL
Insulin Resistance
(Genetic Proxies)
a
b
MASH Liability
(Genetic Instruments)
Total Causal Effect
Illlustrative Findings
(positive causal estimates)
Univariable MR (UVMR)
DAG
Causal effectet
(positive estironates)
Multivariable MR (MVMR)
Hypertension Outcomes
(SBP, DBP, PP)
Positive causal estimates
(independent effects)
4 KEY FINDINGS & INTERPRETATION
MASH
Insulin
IR
High blood pressure
Effect size
1.5
1.0
0.5
0.0
-0.5
ALT MASH Tiers
Effect size
1.0
0.5
0.0
-0.5
-1.0
ALT MASH Tiers
MASH CAUSALLY DRIVES HYPERTENSION
MEDDIATED BY INSULIN RESISTANCE
INDEPENDENT OF CONPOUNDERS (BMI, Lipids)
IMPLICATIONS FOR CARDIOVASCULAR RISK & MASH TREATMENT
